# TC299423, a Novel Agonist for Nicotinic Acetylcholine Receptors

**DOI:** 10.3389/fphar.2017.00641

**Published:** 2017-09-26

**Authors:** Teagan R. Wall, Brandon J. Henderson, George Voren, Charles R. Wageman, Purnima Deshpande, Bruce N. Cohen, Sharon R. Grady, Michael J. Marks, Daniel Yohannes, Paul J. Kenny, Merouane Bencherif, Henry A. Lester

**Affiliations:** ^1^Division of Biology and Biological Engineering, California Institute of Technology, Pasadena, CA, United States; ^2^Department of Biomedical Sciences, Joan C. Edwards School of Medicine, Marshall University, Huntington, WV, United States; ^3^Department of Neuroscience, Icahn School of Medicine at Mount Sinai, New York, NY, United States; ^4^Institute of Behavioral Genetics, University of Colorado, Boulder, Boulder, CO, United States; ^5^Department of Psychology and Neuroscience, University of Colorado, Boulder, Boulder, CO, United States; ^6^Targacept, Inc., Winston-Salem, NC, United States

**Keywords:** nicotine addiction, nicotinic acetylcholine receptors, neuroprotection, electrophysiology, transmitter release, α6β2^∗^, hexahydroazocine, pyrimidine

## Abstract

(E)-5-(Pyrimidin-5-yl)-1,2,3,4,7,8-hexahydroazocine (TC299423) is a novel agonist for nicotinic acetylcholine receptors (nAChRs). We examined its efficacy, affinity, and potency for α6β2^∗^ (α6β2-containing), α4β2^∗^, and α3β4^∗^ nAChRs, using [^125^I]-epibatidine binding, whole-cell patch-clamp recordings, synaptosomal ^86^Rb^+^ efflux, [^3^H]-dopamine release, and [^3^H]-acetylcholine release. TC299423 displayed an EC_50_ of 30–60 nM for α6β2^∗^ nAChRs in patch-clamp recordings and [^3^H]-dopamine release assays. Its potency for α6β2^∗^ in these assays was 2.5-fold greater than that for α4β2^∗^, and much greater than that for α3β4^∗^-mediated [^3^H]-acetylcholine release. We observed no major off-target binding on 70 diverse molecular targets. TC299423 was bioavailable after intraperitoneal or oral administration. Locomotor assays, measured with gain-of-function, mutant α6 (α6L9′S) nAChR mice, show that TC299423 elicits α6β2^∗^ nAChR-mediated responses at low doses. Conditioned place preference assays show that low-dose TC299423 also produces significant reward in α6L9′S mice, and modest reward in WT mice, through a mechanism that probably involves α6(non-α4)β2^∗^ nAChRs. However, TC299423 did not suppress nicotine self-administration in rats, indicating that it did not block nicotine reinforcement in the dosage range that was tested. In a hot-plate test, TC299423 evoked antinociceptive responses in mice similar to those of nicotine. TC299423 and nicotine similarly inhibited mouse marble burying as a measure of anxiolytic effects. Taken together, our data suggest that TC299423 will be a useful small-molecule agonist for future *in vitro* and *in vivo* studies of nAChR function and physiology.

## Introduction

Nicotine is believed to be the primary rewarding and addictive compound in tobacco. It also improves cognition and attention (Newhouse et al., 2012), and may reduce the lifetime risk of developing Parkinson’s disease (PD) (Quik and Wonnacott, 2011). These effects are mediated by binding to nicotinic acetylcholine receptors (nAChRs) in the brain (Picciotto et al., 2008; Srinivasan et al., 2014; Henderson and Lester, 2015). Neuronal nAChRs are pentameric, ligand-gated, cation-selective channels formed from α2 – α10 and β2 – β4 subunits. The heteromeric subtypes contain α and β subunits in a 2:3 or 3:2 (α:β) stoichiometric ratio (Nelson et al., 2003). Neuronal nAChRs are present on presynaptic terminals where they modulate the release of neurotransmitters such as dopamine (Gotti et al., 2006; Dani and Balfour, 2011). The subunit composition of the nAChR subtypes determines their pharmacological and biophysical properties, and partially governs their anatomical and subcellular distribution (Jensen et al., 2005).

β2-containing (β2^∗^) nAChRs are among the most nicotine-sensitive subtypes in the brain, responding to agonist concentrations in the 0.2–0.5 μM range (Matta et al., 2007; Kuryatov and Lindstrom, 2011), and play a crucial role in nicotine reward and reinforcement (Picciotto et al., 1998; Maskos et al., 2005; Walters et al., 2006). Mice with β2 genetic deletions (β2KO) self-administer cocaine (Picciotto et al., 1998), but not nicotine (Picciotto et al., 1998; Maskos et al., 2005; Pons et al., 2008), and β2KO mice fail to show conditioned place preference (CPP) for nicotine (Walters et al., 2006). α4β2^∗^ nAChRs are the most common brain subtype, and play a major role in nicotine reward and reinforcement (Picciotto et al., 1998; Tapper et al., 2004; Pons et al., 2008; McGranahan et al., 2011); however, their widespread distribution (Gotti et al., 2006; Baddick and Marks, 2011) means that they may not be the optimal target for smoking cessation, particularly because their persistent activation may induce depressive behavior (Janowsky et al., 1972).

The CNS expression of α6β2^∗^ receptors is more limited than that of α4β2^∗^ nAChRs. α6β2^∗^ nAChRs are selectively expressed in dopaminergic neurons of the ventral tegmental area (VTA) and substantia nigra pars compacta (SNc) as well as neurons in the retina, superior colliculus (Champtiaux et al., 2003; Mackey et al., 2012; Henderson et al., 2014), medial habenula (Henderson et al., 2014; Shih et al., 2014), and locus coeruleus. α6^∗^ nAChRs are also expressed in the peripheral nervous system where they can interact with P2X receptors (Limapichat et al., 2014; Wieskopf et al., 2015). The localization of α6β2^∗^ nAChRs on dopaminergic neurons suggests they may play a significant role in nicotine reward and contribute to nicotine-mediated neuroprotection against PD (Quik and Wonnacott, 2011). Indeed, selective activation of hypersensitive α6^∗^ nAChRs in transgenic mice by low doses of nicotine is sufficient to establish CPP (Drenan et al., 2012). Moreover, α6KO mice fail to self-administer nicotine, a behavior that can be rescued by selectively re-expressing α6^∗^ nAChRs in the VTA (Pons et al., 2008). Taken together, these results suggest that α6β2^∗^ nAChRs are potential targets for smoking cessation and PD neuroprotection therapeutics.

α-Conotoxin MII (and derivatives) are useful probes for processes mediated by α6^∗^ nAChRs (Azam et al., 2010; Perez-Alvarez et al., 2011; Hone et al., 2012, 2013; McClure-Begley et al., 2012; Marks et al., 2014). Pioneering studies also identified a series of drug-like small molecule α6^∗^ nAChR inhibitors (Zhang et al., 2011). There are also potential, small-molecule α6^∗^-selective agonists that bind to α4β2^∗^ nAChRs and α6β2^∗^ nAChRs (Breining et al., 2009; Marks et al., 2009) or α6β4 nAChRs (Lowe et al., 2010).

Here, we introduce a novel nAChR agonist, (E)-5-(pyrimidin-5-yl)-1,2,3,4,7,8-hexahydroazocine (TC299423). We compare TC299423 to other well-characterized nAChR ligands (nicotine and varenicline) using *in vitro* and *in vivo* assays. The results show that TC299423 is a potent agonist for β2^∗^ nAChRs, and may show a modest preference for α6β2^∗^ over α4β2^∗^ nAChRs. Bioavailability assays show that TC299423 enters the brain. Locomotor assays using transgenic mice expressing hypersensitive α6^∗^ nAChRs confirm that TC299423 potently activates α6^∗^ nAChRs *in vivo*. CPP assays using mice with various nicotinic subunit null mutations further suggest that TC299423 acts primarily through α6(non-α4)β2^∗^ nAChRs. Given that β2^∗^ nAChRs are implicated in the anxiolytic effects of nicotine (Turner et al., 2010; Hone et al., 2013), we use a marble-burying assay to compare the effects of TC299423 to nicotine and varenicline. We also compare the effects of TC299423 to those of nicotine and varenicline for their antinociceptive properties. The results show that TC299423 is a potent nAChR ligand that may useful in the future study of nAChR function and physiology using *in vivo* and *in vitro* models.

## Materials and Methods

### Mice

C57BL/6 mice (ages 3–6 months) used in this study were bred and maintained at the California Institute of Technology (Caltech) or the University of Colorado Boulder. After weaning at 21 days, same sex littermates were housed no more than 4 (Caltech) or 5 (Colorado) to a cage. Mice of the α4 subunit null mutant line (Marubio et al., 1999), the α6 subunit null mutant line (Champtiaux et al., 2002), and the hypersensitive α6L9’S transgenic mice (Drenan et al., 2008) were bred and maintained as above and genotyped as previously described (Marubio et al., 1999; Champtiaux et al., 2003; Drenan and Lester, 2012). Mice had free access to food and water and were maintained on a 12/12-h light/dark cycle at 22°C. All experiments were conducted with the approval of the California Institute of Technology Animal Care and Use Committee. C57BL/6J strain mice, as well as α4, α5, and β2 subunit null mutant mice on this background, were bred and maintained at the Institute for Behavioral Genetics, University of Colorado Boulder, CO, United States. Animal care and procedures were approved by the Animal Care and Utilization Committee of the University of Colorado, Boulder.

### Rats

Rat experiments were conducted in adherence with the National Institutes of Health guidelines and approved by the Institutional Animal Care and Use Committee of Mt. Sinai, New York, NY, United States. Male Wistar rats (*n* = 8; Charles River Laboratories, Raleigh, NC) weighing 300 g at the start of experiments were housed in groups of 1–2 per cage in an environmentally controlled vivarium on a 12 h reverse light-dark cycle. Prior to the commencement of behavioral testing, all rats had *ad libitum* access to food and water.

### Compounds and Reagents

TC299423, (E)-5-(pyrimidin-5-yl)-1,2,3,4,7,8-hexahydroazocine, was synthesized at Targacept. A full report on the synthesis of TC299423 is in preparation. Varenicline tartrate was also synthesized by Targacept. [^125^I]-Epibatidine (2200 Ci/mmol), [^3^H]dopamine (3,4-[ring-2,5,6-3H], 30–60 Ci/mmol), [^3^H]choline (methyl-3H, 60–90 Ci/mmol), and carrier-free ^86^RbCl were purchased from Perkin Elmer Life Sciences, Boston, MA, United States. α-Conotoxin MII (α-CtxMII) was obtained from J. Michael McIntosh, University of Utah, Salt Lake City, UT, United States. The following chemicals as well as all buffer components (Reagent Grade) were products of Sigma–Aldrich (St. Louis, MO, United States): (L)-nicotine hydrogen tartrate, mecamylamine, atropine, bovine serum albumin (BSA), (±)-epibatidine, HEPES, nomifensine, pargyline, and tetrodotoxin. All compounds were dissolved in physiological saline (0.9% NaCl). Concentrations refer to the free base.

### Biochemistry and Physiology

#### Ligand Binding

The methods used for preparing brain membranes in hypotonic buffer have been described previously (Marks et al., 1998, 2007). Brain membrane preparations were stored as pellets under buffer at -70°C or used immediately. We followed previously published methods for [^125^I]epibatidine binding and analysis (Grady et al., 2010).

#### Synaptosome Preparation

Regions of interest were dissected from fresh mouse brains and homogenized in ice-cold isotonic sucrose (0.32 M), buffered with HEPES (5 mM, pH 7.5). The suspension was centrifuged at 12,000 × *g* for 20 min. The pellet was re-suspended in the appropriate uptake buffer (Grady et al., 2001; Salminen et al., 2004; Marks et al., 2006) and used immediately.

#### [^3^H]Dopamine Uptake and Release

Superfusion was carried out at 22°C using a buffer containing: NaCl, 128 mM; KCl, 2.4 mM; CaCl_2_, 3.2 mM; MgSO_4_, 1.2 mM; KH_2_PO_4_, 1.2 mM; HEPES, 25 mM; glucose, 10 mM; ascorbic acid, 1 mM; pargyline, 0.01 mM; 0.1% BSA; nomifensine, 1 μM (to prevent the re-uptake of dopamine); atropine, 1 μM (to prevent muscarinic receptor activation); pH 7.5. Superfusion proceeded at 0.7 mL/min for 10 min before stimulation with agonist for 20 s. Selected aliquots were perfused with α-CtxMII (50 nM) for 3 min immediately before stimulation. This concentration of α-CtxMII was sufficient to inhibit α6β2^∗^-nAChRs in the mouse striatum (Salminen et al., 2004, 2007). Fractions (∼0.1 mL) were collected into 96-well plates at 10 s intervals for 4 min (starting ∼1 min before stimulation). After adding 0.15 mL of Optiphase SuperMix scintillation cocktail, radioactivity was measured using a 1450 MicroBeta TriLux counter (Perkin Elmer Life Sciences).

Previously published methods for [^3^H]ACh release from crude IPN synaptosomes were followed (Grady et al., 2001). Agonist-stimulated ^86^Rb^+^ efflux from synaptosomes was measured using previously described methods (Marks et al., 1999, 2007).

#### Neuro-2a Cell Culture

Mouse neuroblastoma 2a (neuro-2a) cells, obtained from ATCC (Cat #: CCL-131, purchased: January 2012), were cultured using previously described methods (Xiao et al., 2011). The β2_DM_ subunit bears mutations in the M3–M4 intracellular loop that enhance exit from the endoplasmic reticulum for both α4β2 and α6β2 nAChRs (Srinivasan et al., 2011; Xiao et al., 2011; Henderson et al., 2014). Cells were transfected with 500 ng of: α4-GFP and β2_DM_ or α6-GFP, β2_DM_, and β3 nAChR subunits. Plasmids were mixed with 250 μL of Opti-MEM reduced serum medium (Life Technologies). Lipofectamine 2000 (Life Technologies) was separately added to 250 μL of Opti-MEM. After 5 min at 24°C, DNA and Lipofectamine solutions were combined and incubated for another 25 min at 24°C. The resulting solution was added to pre-plated neuro-2a cells and incubated for 24 h. After 24 h, the Opti-MEM was removed and replaced with culture medium.

#### Patch-Clamp Recordings

For the patch-clamp experiments, we used mouse neuro-2a cells. 50,000 neuro-2a cells (transfected as described above) were plated onto sterilized 12 mm glass coverslips (Deckgläser, Prague, Czechia), placed in 35-mm culture dishes, and cultured in a humidified incubator (37°C, 95% air, 5% CO_2_). Transfected cells displaying eGFP fluorescence were identified for patching using an inverted epifluorescence microscope. The pipette solution contained (in mM): 135 K gluconate, 5 KCl, 5 EGTA, 0.5 CaCl_2,_ 10 HEPES, 2 Mg-ATP, and 0.1 GTP (pH adjusted to 7.2 with Tris-base, osmolarity adjusted to 280–300 mOsm with sucrose). The resistance of the patch pipettes was 2–4 MΩ. The extracellular solution contained (in mM): 140 NaCl, 5 KCl, 2 CaCl_2_, 1 MgCl_2_, 10 HEPES, and 10 glucose (320 mOsm, pH set to 7.3 with Tris-base). The cells were voltage-clamped in whole-cell mode using an Axopatch 1D amplifier (Axon Instruments), and the data were recorded with a Digidata 1440A analog-to-digital converter (Axon Instruments) and the pCLAMP V.10 software (Axon Instruments). The data were sampled at 10 kHz and low-pass filtered at 2 kHz prior to digitization. All experiments were performed at 24°C and the recording chamber was continually perfused with extracellular solution. To avoid receptor desensitization by repetitive agonist application, agonists were applied at intervals ≥3 min. An Octaflow II superfusion system (ALA Scientific Instruments) was used to apply the agonist for concentration-response studies. Applications were 500 ms at 6 psi. For the generation of concentration response curves, test pulses were applied to assess functional run-down (three pulses at 0.5 μM TC299423 or 1.0 μM TC299423 for α6-GFPβ2_DM_β3 and α4β2_DM_ nAChRs, respectively). Following the collection of the full concentration range an additional test pulse was repeated to assess accumulated desensitization.

#### Data Analysis for Patch-Clamp Assays

Data were fit to a single Hill term using Origin 10 software. Data are expressed as mean ± SEM. The SEM values were calculated from the EC_50_ values obtained for each individual concentration-response obtained for α6-GFPβ2β3 or α4-GFPβ2 nAChRs (*n* => 5 and 4, respectively). For both α6-GFPβ2β3 and α4-GFPβ2 nAChRs we used peak current amplitude, normalized to the response of 1 μM TC299423.

### Behavioral Assays

For the mouse behavioral assays all drugs were dissolved in sterile 0.9% saline and were administered in an injection volume of 0.1 ml/g. Drug doses are expressed as free base.

### Locomotor Assays

Mice were habituated to the experimental room for 2 h prior to the experiment. Horizontal locomotor activity was recorded using an infrared photobeam activity cage system (San Diego Instruments; San Diego, CA, United States). After habituating to the experimental room, mice were placed singly in a novel cage. Ambulations were recorded when two contiguous photobeams were broken in succession, preventing activity from being recorded by most sedentary animals. Ambulation events were measured for 45 min with four 15 s intervals per min. Mice were given a total of two intraperitoneal (*ip*) injections. First, mice were pre-injected with saline or 1 mg/kg mecamylamine before being placed in the activity cages. After 8 min in the cage they were removed, injected with the second drug (saline, nicotine, or TC299423), and returned again to the cage for the 45 min session.

#### Conditioned Place Preference

Male, 3–6-month-old mice were used in our CPP assays. Various genotypes were used: WT, α6L9′S, α4L9′S (hypersensitive α4^∗^ and α6^∗^ nAChRs, respectively), α6KO, α4KO, and β2KO. All WT mice were C57BL/6J and all other genotypes have been backcrossed with C57BL/6J mice for ≥10 generations. The CPP apparatus (Med Associates, East Fairfield, VT, United States) was a rectangular cage with interior dimensions of 46.5 × 12.7 × 12.7(H) cm, divided into three sub-compartments: white and black (each 16.8 cm L) with steel mesh and rod floors, respectively, and a central gray compartment (7.2 cm L) with a solid plastic floor. Each compartment had a polycarbonate hinged lid for loading the animals. Drop-down doors separated the chambers.

Mice were housed singly and habituated to the experimental room for 3–7 days before initial testing and remained in the experimental room for the duration of the experiment. We chose a biased method for CPP. On day 1 (pre-training) mice were placed in the center chamber and allowed to explore the apparatus freely for 20 min. Time spent in each chamber was recorded, and drug pairing was determined by the least preferred chamber. Mice with a severe initial bias for one chamber (defined as ≥65% time spent in one conditioning chamber) were excluded. On days 2, 4, 6, and 8, mice were injected with the drug of interest, and were confined to the drug-paired chamber for 20 min. On days 3, 5, 7, and 9, mice were injected with saline and confined to the opposite chamber. On day 10 (post-training), mice were again given free access to the apparatus for 20 min, and the time spent in each chamber was recorded. CPP was determined by measuring the change in the difference between the time spent in the drug-paired chamber and the saline-paired chamber from pre- to post-training.

#### Marble Burying Assay

Mice were habituated to the experimental room for ≥2 h prior to testing. Fifteen marbles were placed approximately 5 cm apart in a 5 × 3 marble grid in an activity cage, with 5 cm-deep bedding. Mice were given *ip* injections of saline, nicotine, varenicline, or TC299423 and placed in the cage with the marbles. After 10 min, the mice were returned to their home cages. Marbles were counted as buried if they were at least 75% covered with bedding. Each mouse was tested under each experimental condition using a Latin-square crossover design. A day without testing occurred between each test.

#### Hot Plate Assay

Mice were again habituated to the experimental room for ≥2 h prior to testing. Mice were given *ip* injections of saline, nicotine, varenicline, or TC299423, 5 min before being placed on the hot plate apparatus (Harvard Apparatus, Holliston, MA, United States). This apparatus is a heated metal plate, maintained at 55°C, enclosed by a clear acrylic plastic cylinder (∼10 cm in diameter), within which the mouse is free to move. When a mouse exhibits evidence of discomfort (such as paw shaking, paw licking, jumping, or vocalization) or when a cut-off time of 60 s is reached, it is removed from the apparatus. If the mouse urinates during the assay, it is immediately removed from the hot plate. The time that the mouse remains on the hot plate prior to showing signs of discomfort is recorded. Doses were administered using a Latin-square crossover design. A day without testing occurred between each test.

#### Rat Intravenous Self-Administration

Operant chambers (Med Associates, East Fairfield, VT, United States) were used for food delivery and the intravenous self-administration (IVSA) of nicotine. The chambers were equipped with two response levers (usually designated active and inactive) with a cue light located above each lever, a food pellet dispenser located between the levers, and a computer-controlled injection pump for the scheduled delivery of nicotine via an IV implanted catheter.

For IV catheter implantation, the rats were anesthetized using 1–3% isoflurane inhalation in oxygen and surgically prepared with catheters in the left jugular vein (Kenny et al., 2008). The catheter was passed subcutaneously to a polyethylene assembly mounted on the animals’ back. Prior to training, rats were food-restricted so that their body weight was approximately ∼85% that of free-feeding rats. They were then trained to press an active lever for 45 mg food pellets on a fixed ratio 5 time-out 20 s (FR5TO20) schedule of reinforcement. An inactive lever was also present in the operant box. Pressing this lever was recorded but was not associated with scheduled consequence. Rats were permitted to respond for food rewards until a reliable response was achieved, defined as >90 pellets earned per 1 h session for three consecutive sessions. Rats were then permitted to respond for food (*n* = 8) or for nicotine infusions (*n* = 9) during 1 h daily sessions under the FR5TO20 schedule. Each food reward (45 mg pellet) or nicotine reward of 0.19 μmol/kg (0.03 mg/kg) per infusion over 1 s initiated a 20 s time-out period, signaled by a light cue located above the active lever, during which time pressing the active lever was without consequence.

Rats received *ip* injections of TC299423 (injection volume of 1 mL per 300 g weight). Twenty min after injection, rats were placed in the operant boxes and responding for food rewards or nicotine infusions was recorded. After each session, catheters were flushed with heparinized saline (30 U per mL) and checked for leaks or blockages. The effects of vehicle, or TC299423 doses of 0.01–0.08 mg/kg, on food or nicotine responding were assessed using a within-subjects Latin-square design. Each rat was permitted to respond for food or nicotine for at least two IVSA sessions between each TC299423 treatment to allow responding to return to baseline levels.

### Off-Target Binding and Pharmacokinetic Data

#### Pharmacological Profiling

NovaScreen assays at PerkinElmer – Caliper Life Sciences (Waltham, MA, United States) were used to evaluate the affinity of TC299423 on 70 diverse molecular targets *in vitro* using competition radioligand binding assays (see Supplementary Table S3).

#### Pharmacokinetics

Pharmacokinetic experiments with TC299423 were performed by Absorption Systems LP (Exton, PA, United States). Eighteen male and female mice (20–40 g) were administered TC299423 at an *ip* dose of 0.3 mg/kg. Blood samples were taken and the brains were harvested 5 min, 15 min, 30 min, 1 h, 3 h, and 6 h after drug administration. Fifteen male and female mice (20–40 g) were administered TC299423 at a dose of 1 mg/kg orally. Blood and brain samples were obtained at the same time points as above. Untreated plasma and blood samples were collected from three mice in the same cohort as the study animals for pre-dose (time 0) samples. Blood samples were collected by cardiac puncture and stored in tubes containing sodium heparin before being processed. Plasma was prepared and frozen for analysis. Brains were harvested, rinsed, patted dry, weighed, and frozen.

The concentration of TC299423 in brain and plasma samples was measured using a generic LC-MS/MS method with a minimum 6-point calibration curve. Matched matrix was used for preparation of calibration standards. Dosing solutions were normalized in matched matrix and analyzed in triplicate in the same analytical batch as the incurred samples.

#### Radioligand Displacement

Unbound TC299423 concentrations in the brain and blood of C57BL/6 mice were also measured using a radioligand displacement assay (Hussmann and Kellar, 2012). Mice were administered 0.2 mg/kg (free base) TC299423 *ip*, and euthanized 5, 10, or 20 min after drug injection. Uninjected mice served as controls (*n* = 3 for each group). Trunk blood was collected into heparinized 1.5 mL polyethylene tubes. The tubes were centrifuged and the serum was collected. Some hemolysis occurred. The cerebral cortex was dissected, placed in 1 mL of cold water, homogenized, and diluted to 3 mL with cold water. Epibatidine saturation curves were constructed for the cortical samples using eight concentrations of [^125^I]epibatidine measured in triplicate (6, 12, 25, 50, 100, 200, 400, and 800 pM). The final assay volume was 500, and 50 μL of homogenate was added to each sample. Blanks included 100 μM nicotine. Samples were incubated at room temperature overnight. The samples were filtered, washed and counted. Inhibition of [^125^I]epibatidine (325 pM) binding was measured by adding 1, 2, 5, 10, or 20 μL of serum to samples. The washed, particulate fraction from C57BL/6 cortex was the source of the binding sites.

## Results

### Identification of TC299423

**FIGURE 1:**
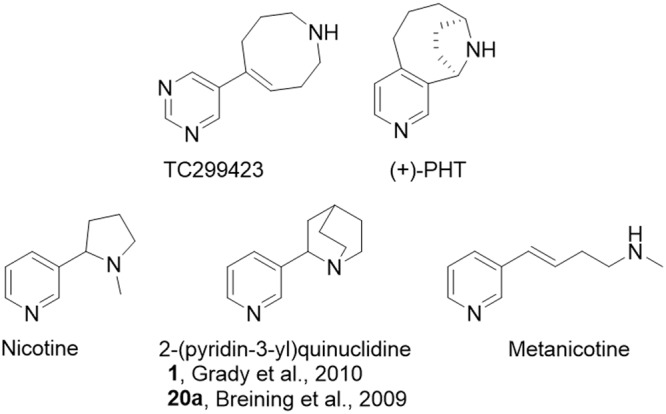
Relevant chemical structures. Structures for TC299423 (189 g/mol), (+)-PHT (molecular mass, 188 g/mol) which has also been tested as a potential α6^∗^ selective compound [62], nicotine (162 g/mol), 2-(pyridine-3-yl)quinuclidine (188 g/mol), a previous potential lead molecule from a series of metanicotine analogs (Breining et al., 2009; Grady et al., 2010), and metanicotine (162 g/mol).

In preparation for the present study, a small-molecule discovery program using a library of ∼7000 compounds of modest drugability was conducted at Targacept. Initial screenings of the library were conducted with few-concentration assays of Ca^2+^ flux and nAChR binding in transfected cell lines. Molecules with potential nanomolar to micromolar affinity for α6β2^∗^ nAChRs were sought. TC299423 (**Figure 1**), one of these compounds, was identified after our previous structure-activity relationship (SAR) studies on other nAChR subtypes (Breining et al., 2012; Zhang et al., 2012; Strachan et al., 2014) and on α6β2^∗^ nAChRs (Breining et al., 2009; Grady et al., 2010; Strachan et al., 2014). TC299423 was also analyzed in computational studies on homology models of α6β2 binding sites analogous to those described previously (Strachan et al., 2014). Assays that assessed metabolism were also conducted (discussed below, see Supplementary Table S2). These initial experiments motivated the more detailed studies in the present paper, and in recently reported structure-function experiments (Post et al., 2015; Post et al., 2017).

TC299423 can be considered an acrylic metanicotine analog (1 or 20a, **Figure 1**). In TC299423, the pyridine ring of nicotine was replaced by a pyrimidine ring, a substitution previously shown to increase α6^∗^ selectivity (Breining et al., 2009). We also incorporated the *N*-methyl moiety of the metanicotine into an additional, 8-membered ring (a 1,2,3,4,7,8-hexahydroazocine) in an effort to test a hypothesis that conformational constraints imposed by a cyclic structure would confer additional selectivity by reducing the conformational flexibility of the linear metanicotine structure.

Data obtained with an oocyte expression system showed that TC299423 exhibits a binding mode similar to that of nicotine at α6β2 nAChRs, forming a relatively weak cation-π interaction with a conserved tryptophan residue termed TrpB (Post et al., 2015). At α4β2 nAChRs, this cation-π interaction also occurs. At α4β2 nAChRs, TC299423, like several other secondary ammonium agonists, makes an additional cation-π interaction with a conserved tyrosine termed TyrC2 (Post et al., 2017).

### Pharmacokinetics

**FIGURE 2:**
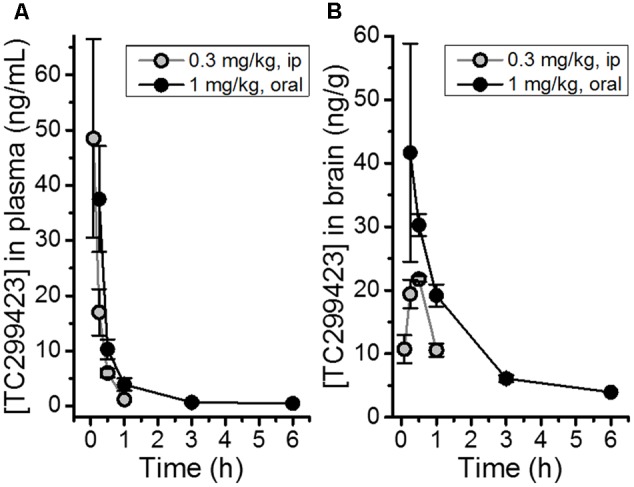
Pharmacokinetic measurements for TC299423. **(A)** Concentrations of TC299423 in plasma (ng/mL) following ip (0.3 mg/kg) and oral (1 mg/kg) administration. **(B)** Concentrations of TC299423 in the brain (ng/g) following ip (0.3 mg/kg) and oral (1 mg/kg) administration. All data are mean ± SEM. *n* = 3 mice. See text for conversion of ng/ml to μM.

The bioavailability of TC299423 was studied by measuring its plasma and brain concentration in mice after *ip* or oral administration (**Figure 2**). TC299423 reached a maximum plasma concentration of 49 ± 18 ng/ml (0.26 μM) 0.08 h after a 0.3 mg/kg *ip* injection and had a half-life of 0.17 h in the plasma (**Figure 2A**). It reached a maximum brain-tissue concentration of 22 ± 0.43 ng/g (0.12 μM) 0.5 h after *ip* injection (**Figure 2B**). Oral administration at 1 mg/kg resulted in a maximum plasma concentration of 38 ± 10 ng/ml (0.20 μM) after 0.25 h with a half-life of 1.12 h in the plasma (**Figure 2A**). A maximum brain-tissue concentration of 42 ± 17 ng/g (0.22 μM) was attained at 0.25 h (**Figure 2B**). Thus, TC299423 enters the plasma, and passes into the brain, before being completely metabolized. We used a previously described radioligand displacement assay (Hussmann and Kellar, 2012) to obtain additional measurements of the TC299423 concentration in brain tissue *ex vivo* (Supplementary Table S1). Similar to the previous pharmacokinetic assay, these data confirm the penetration of TC299423 into the brain and its availability to nAChRs, for ≥20 min.

Some aspects of the microsomal metabolism of TC299423, in comparison to varenicline, were studied in preliminary experiments. In both human and rat microsomes, TC299423 exhibited a ∼7-fold longer half-life compared to varenicline (Supplementary Table S2). Using recombinantly expressed cytochrome P450 enzyme preparations, we also observed that TC299423 exhibits a more diverse degradation pathway than varenicline as it is metabolized by more isoforms of cytochrome P450 enzyme (Supplementary Table S2). These data suggest that several metabolic pathways contribute to TC299423 degradation. Thus, it is a candidate for more detailed human metabolism studies.

### Potency and Efficacy of TC299423

**FIGURE 3:**
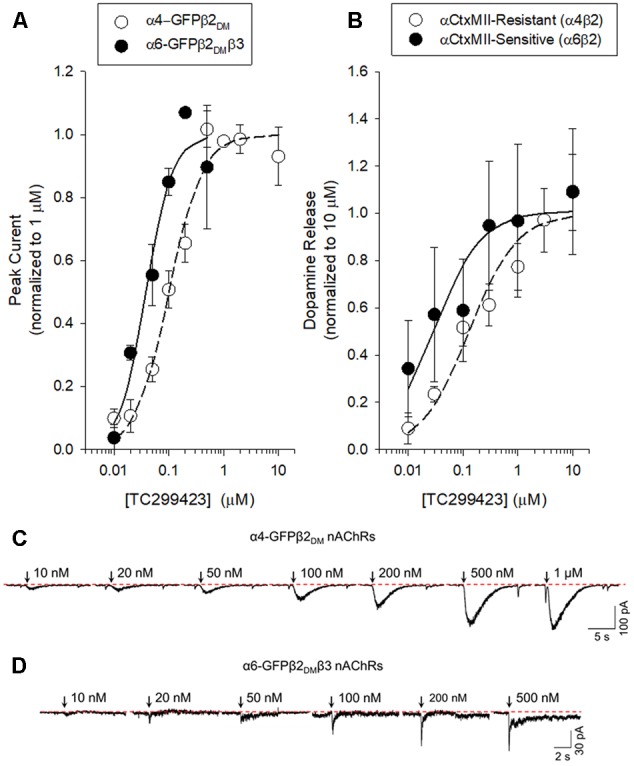
Concentration-response curves for functional responses of TC299423 on nAChRs. **(A)** Concentration-response relations of TC299423 on α4-GFPβ2_DM_ nAChRs (*n* = 5–13 cells) and α6-GFPβ2_DM_β3 nAChRs (*n* = 4 cells). **(B)** Measurement of α4β2^∗^-mediated α-CtxMII-resistant [^3^H]-dopamine release and α6β2^∗^-mediated (α-CtxMII-sensitive) [^3^H]-dopamine release from WT mouse striatal synaptosomes (see Materials and Methods). EC_50_ values for **(A,B)** are given in **Table 1**. Data for [^3^H]-dopamine release were normalized to the maximum response calculated from the non-linear curve fits of the results to the Michaelis–Menten equation. Maximal response for the α-CtxMII-resistant release was calculated to be 6.06 ± 0.37 units and maximal response for the α-CtxMII-sensitive release was 0.93 ± 0.08 units. For comparison, 10 μM nicotine elicited 7.78 ± 0.83 units for α-CtxMII-resistant release and 3.63 ± 0.81 units for α-CtxMII-sensitive release. Data are mean ± SEM. SEM values for the normalized data were calculated using Taylor’s expansion. Additional data are presented in the Supplementary Figure S1. **(C,D)** Representative waveforms of voltage-clamp currents for TC299423 applications at given concentrations on α6-GFPβ2_DM_β3 and α4-GFPβ2_DM_ nAChRs. Waveforms are from a single cell for each nAChR subtype. Arrows indicate 500 ms applications and the dotted red line represents the baseline for measurements.

We used a variety of assays (whole-cell patch clamp, synaptosomal [^3^H]-dopamine release, synaptosomal [^3^H]-ACh release, [^125^I]-epibatidine binding, and ^86^Rb^+^ efflux) to measure TC299423 concentration-response relations for α4β2^∗^, α6β2^∗^, and α3β4^∗^ nAChRs. **Figure 3, Tables 1–3**, and Supplementary Figure S1, summarize the data and show that TC299423 potently activates α6β2^∗^ nAChRs. The TC299423 concentration-response relations for α4β2 and α6β2β3 nAChRs were measured by transiently transfecting fluorescently tagged, mutant α4-GFPβ2_DM_ or α6-GFPβ2_DM_β3 nAChRs in neuro-2a cells, voltage clamping the cells (whole-cell mode), and applying TC299423 by rapid microperfusion (see Materials and Methods, **Figure 3A**). GFP-labeled nAChR subunits were used to facilitate the identification of neuro-2a cells expressing the transfected nAChRs and the DM mutation was used to increase the surface receptor density (Henderson et al., 2014). EC_50_ values were determined by fitting the concentration-response data to the Hill equation. The EC_50_ of TC299423 on α4-GFPβ2_DM_ nAChRs was 0.1 ± 0.02 μM and on α6-GFPβ2_DM_β3 nAChRs was 0.04 ± 0.01 μM (**Figure 3A** and **Table 1**). The EC_50_ for the α6-GFPβ2_DM_β3 nAChRs was significantly less than that for α4-GFPβ2_DM_ nAChRs (*p* < 0.05, two-tailed *t*-test). Thus TC299423 activated α6-GFPβ2_DM_β3 nAChRs significantly more potently than α4-GFPβ2_DM_ nAChRs.

**Table 1:** EC_50_ values and Efficacy of TC299423 on nAChRs.

Assay	Transfected nAChR Subunits in Neuro-2a Cells				
	α6β2β3	α4β2 Single component			
**Patch clamp** EC_50_ (μM)	0.04 ± 0.01	0.10 ± 0.02			

	**Probable nAChR subunit composition in mouse brain**				
**Neurotransmitter release**	**α6β2^∗a^**	**α4β2^∗ a^ Single component**	**α3β4^∗b^**	**α6β2^∗a^ (α5KO)**	**α4β2^a^ (α5KO)**

% Efficacy EC_50_ (μM)	50 ± 20% 0.03 ± 0.01	76 ± 5% 0.13 ± 0.03	100 ± 16% 8.0 ± 0.4	54 ± 14% 0.05 ± 0.03	112 ± 9% 0.6 ± 0.1

*^a^Data collected from [^*3*^H]DA release, striatum. ***^b^***[^*3*^H]ACh release, interpeduncular nucleus TC299423 EC_*50*_ and efficacy on nAChRs transfected into neuro-2a cells or with endogenous α4β2^∗^, α6β2^∗^, and α3β4^∗^-nAChRs in mouse brain tissue. Efficacy values are given as a percent of the response to nicotine, 10 μM. EC_50_ data are expressed as mean ± SEM. See **Figure 3** and Supplementary Figure S1 for full concentration-response curves.*

**Table 2:** Efficacy and Potency of TC299423 on stoichiometries of α4β2 nAChRs.

	α4β2^∗^ Two components		α4β2 (α5KO)
	High-sensitivity α4_(2)_β2_(3)_	Low-sensitivity α4_(3)_β2_(2)_	
**^86^Rb^+^ efflux, Thalamus**			
Efficacy	139 ± 14%	32 ± 10%	166 ± 32%
EC_50_ (μM)	0.6 ± 0.1	10 ± 10	1.6 ± 0.5
**^86^Rb^+^ efflux, Cortex**			
Efficacy	94 ± 7%	39 ± 4%	169 ± 20%
EC_50_ (μM)	2 ± 2	26 ± 21	2.4 ± 0.7

*Efficacy values are given as a percent of the response to nicotine, 10 μM. EC_*50*_ data are expressed as mean ± SEM. See Supplementary Figure S1 for full concentration-response curves.*

**Table 3:** Binding *K*_i_ of TC299423 on nAChRs.

	α4β2^∗^ Single component	α6β2^∗^ (α4KO)	α3β4^∗^ (β2KO)
**[^125^I]epibatidine binding**			
K_i_ (nM)	0.24 ± 0.04	1.4 ± 0.6	18 ± 0.7

*Data are expressed as mean ± SEM.*

To compare the efficacy and potency of TC299423 for native α4β2^∗^ and α6β2^∗^ nAChRs, we measured TC299423-induced [^3^H]dopamine release from striatal synaptosomes (**Figure 3B** and **Table 1**). Two classes of nAChR subtypes are responsible for nicotine-induced [^3^H]dopamine release from striatal synaptosomes: α-conotoxin MII (α-CtxMII)-sensitive and α-CtxMII-insensitive nAChRs. These classes are composed of α6β2^∗^ and α4(non-α6)β2^∗^ nAChRs, respectively. Accordingly, we measured concentration-response relations for the α-CtxMII-sensitive, and -insensitive, TC299423-induced [^3^H]dopamine release. As above, the EC_50_ values were obtained from fits to the Hill equation. The EC_50_ of TC299423 for α-CtxMII-insensitive [^3^H]dopamine release was 0.13 ± 0.03 μM and that for α-CtxMII-sensitive release was 0.03 ± 0.01 μM (**Figure 3B** and **Table 1**). Thus, consistent with the patch-clamp data above, the TC299423–induced [^3^H]dopamine release assay also suggests that it activates α6β2^∗^ nAChRs more potently than α4β2^∗^ nAChRs. Both data sets suggest that TC299423 activates α6β2^∗^ nAChRs ∼3-fold more potently than α4β2^∗^ nAChRs. We also studied the effects of TC299423 on [^3^H]dopamine release using striatal synaptosomes from α5-KO mice. Genetic deletion of the α5 subunit did not significantly affect the potency of TC299423-induced α-CtxMII-sensitive, or -insensitive, [^3^H]dopamine release (**Table 1**).

To study the potency of TC299423 for native α3β4^∗^ nAChRs, we prepared synaptosomes from the mouse interpeduncular nucleus, a region with dense α3β4 nAChR expression (Shih et al., 2014), and measured TC299423-induced [^3^H]ACh release. The EC_50_ for TC299423-induced [^3^H]ACh release was 8.0 ± 0.4 μM (**Table 1**). Thus, TC299423 was much less potent at eliciting [^3^H]ACh release from interpeduncular nucleus synaptosomes than it was at eliciting [^3^H]dopamine release from striatal synaptosomes, suggesting that the drug activates α6β2^∗^ and α4β2^∗^ nAChRs more potently than α3β4^∗^ nAChRs.

The α4 and β2 nAChR subunits can form functional nAChRs with different subunit stoichiometries [α4_(3)_β2_(2)_, α4_(2)_β2_(3)_] and agonist sensitivities (low-, high-sensitivity, respectively). Receptors with the α4_(3)_β2_(2)_ stoichiometry are much less ACh-sensitive than those with the α4_(2)_β2_(3)_ stoichiometry (Nelson et al., 2003; Kuryatov et al., 2005). To compare the efficacy and potency of TC299423 for these two receptor stoichiometries, we measured TC299423-elicited ^86^Rb^+^ efflux from mouse thalamic and cortical synaptosomes (**Table 2**). The α4(non-α6)β2^∗^ nAChRs mediate nicotinic agonist-induced ^86^Rb^+^ efflux from these synaptosomes (Marks et al., 2009). The nicotinic antagonist dihydro-β-erythroidine (DhβE) blocks high-sensitivity α4β2^∗^ nAChRs more potently than low-sensitivity α4β2^∗^ nAChRs (Marks et al., 1999) and can be used to distinguish between the two receptor stoichiometries. TC299423 had an EC_50_ of 0.6–2.0 μM for the DhβE-sensitive α4β2^∗^ nAChRs and ≥14 μM for the DhβE-insensitive α4β2^∗^ nAChRs (**Table 2**). Thus, TC299423 more potently activates high-sensitivity α4_(2)_β2_(3)_, than low-sensitivity α4_(3)_β2_(2)_, nAChRs.

Regarding efficacy, previous data with an oocyte expression system showed that TC299423 is a partial agonist at α6β2 nAChRs, with an efficacy 59% that of ACh itself (Post et al., 2015). In the present experiments on neurotransmitter release, we found additional evidence that TC299423 is a partial agonist at α6β2^∗^ receptors: the maximal release by TC299423 was 50–54% that of nicotine (**Table 1**).

We find that TC299423 is roughly as efficacious as nicotine for high-sensitivity α4β2^∗^ nAChRs, but a partial agonist on low-sensitivity α4β2^∗^ nAChRs (**Tables 1, 2**). Interestingly, it also appears to be at least as efficacious as nicotine for α4β2^∗^ nAChRs lacking the α5 nAChR subunit (**Figure 1, Tables 1, 2**, and Supplementary Table S1). We also find that TC299423 is roughly as efficacious as nicotine for α3β4^∗^ nAChRs.

### Binding Affinity of TC299423

To determine whether TC299423 displays a higher affinity for β2^∗^, than β4^∗^, nAChRs, we measured its ability to displace [^125^I]epibatidine binding to membranes from three brain regions (see Materials and Methods). [^125^I]Epibatidine binds predominantly to the α4β2^∗^ subtype in cortical membranes, α3β4^∗^ in IPN membranes from β2KO mice, and α6β2^∗^ in striatal membranes from α4KO mice (Grady et al., 2010). Thus, the set of *K*_i_ values for TC299423 to displace [^125^I]epibatidine binding in these three regions provides a way to measure the apparent affinity of these three subtypes. Based on these measurements, the *K*_i_ values for the α4β2^∗^, α6β2^∗^, and α3β4^∗^ subtypes were 0.24 ± 0.04, 1.4 ± 0.6, and 18.0 ± 0.7 nM, respectively (**Table 3**). Thus, TC299423 binds with higher affinity to α4β2^∗^ and α6β2^∗^, than α3β4^∗^ nAChRs. *K*_i_ values primarily reflect agonist binding to the high-affinity, desensitized state, whereas EC_50_ values for functional assays reflect agonist binding to the free-receptor state. The relationship between the *K*_i_ for [^125^I]epibatidine displacement (**Table 3**) and the EC_50_ for [^3^H]dopamine release (**Table 1**) for the α4β2^∗^ and α6β2^∗^ nAChRs is consistent with previous results showing that α6^∗^ nAChRs display a ∼10-fold lower *K*_i_ than EC_50_, whereas α4^∗^ nAChRs display a ∼100-fold difference. Here, we observed a ∼20-fold difference for α6β2^∗^ nAChRs and a ∼500-fold difference for α4β2 nAChRs. This comparison suggests that agonist-induced, steady-state desensitization is less pronounced for α6β2^∗^, than α4β2^∗^, nAChRs (Grady et al., 2012).

### TC299423 Activity on Non-nAChR Targets

TC299423 was assessed for binding to 70 different receptors and other targets at a concentration of 1 μM (Supplementary Table S3). Assays included the nicotinic drug [^3^H]epibatidine binding to α-bungarotoxin-insensitive nAChRs, the 5-HT3 inhibitor [*N*-*methyl*-^3^H]GR65630 to mouse receptors in N1E-115 cells, and human receptors expressed in a clonal cell line. Other assays included monoamine oxidase A, monoamine oxidase B, 5-HT transporter, norepinephrine transporter, and dopamine transporter, as well as other. G-protein-coupled receptors, ion channels, enzymes, and transporters. The results showed that, consistent with the results reported here, TC299423 bound to neuronal nAChRs (measured by [^3^H]epibatidine displacement). It also bound to ATP-sensitive potassium channels, but with a much lower affinity than to nAChRs. Only these two sites showed significant binding. Thus, off-target effects are unlikely to confound our experimental data at TC299423 concentrations ≤1 μM.

### TC299423 and Reward-Related Behavior

**FIGURE 4:**
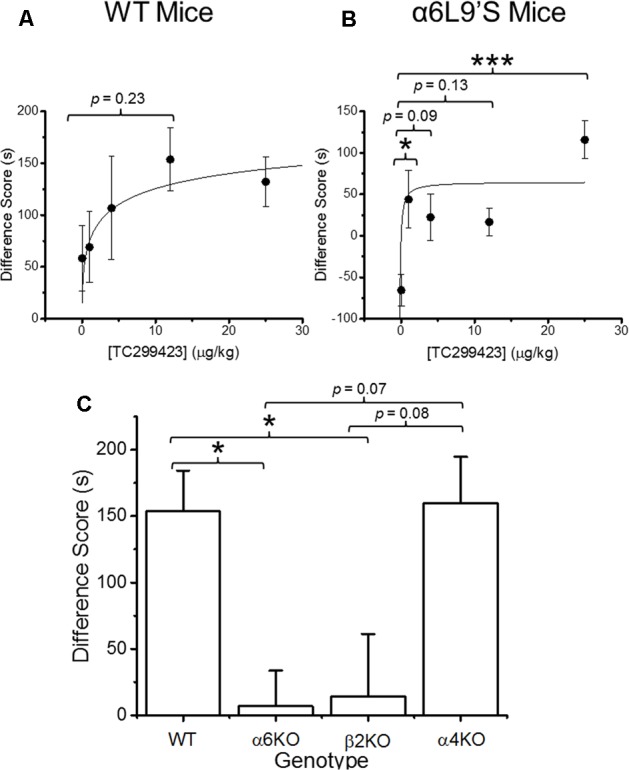
Conditioned place preference (CPP) by genotype and dose. **(A)** WT mice were trained with TC299423 using a range of doses (0, 1, 4, 12, and 25 μg/kg) in a CPP assay (*n* = 8–23). A Michaelis–Menten curve was fitted to the data. The ED_50_ was 4 ± 3 μg/kg. **(B)** α6L9′S mice were trained in a CPP assay using the same doses as in **(A)** (*n* = 8–10). A Michaelis–Menten curve is drawn on the data with an ED_50_ of 0.3 ng/kg to guide the eye; but the data would be equally well described by any ED_50_ < 1 ng/kg. **(C)** Mice of various genotypes were trained with TC299423 (12 μg/kg) in a CPP assay (*n* = 9–23 mice). For all panels, data shown are mean ± SEM: ^∗^*p* < 0.05, ^∗∗^*p* < 0.01, ^∗∗∗^*p* < 0.001 (one-way ANOVA with *post hoc* Tukey).

Previous data show that mutant mice expressing hypersensitive α6L9′S^∗^ nAChRs are more sensitive to nicotine-induced CPP than WT mice, suggesting that α6^∗^ nAChR activation on its own can induce CPP (Drenan et al., 2012). Our functional data show that TC299423 exhibits a modest preference for activating α6^∗^ nAChRs. Thus, CPP was used to test whether the activation of α6^∗^ nAChRs by TC299423 is rewarding in mice (**Figure 4**). We tested the ability of TC299423 to establish CPP in both WT, and mutant α6L9′S, mice. Mice were injected with zero (saline-injected), 1, 4, 12, or 25 μg/kg TC299423 during CPP training (see Materials and Methods). TC299423 had a highly significant overall effect on the CPP difference score in the mutant α6L9′S mice [One-way ANOVA, *F*_(4,45)_ = 6.706, *p* = 0.0003], but not in WT mice [One-way ANOVA, *F*_(4,60)_ = 1.442, *p* = 0.2312] (**Figures 4A,B**). *Post hoc* comparisons (Tukey HSD) showed that the scores of α6L9′S mutant mice injected with 1 and 25 μg/kg TC299423 were significantly greater than the saline-injected controls (*p* < 0.05, **Figure 4B**). The ability of low doses of TC299423 to elicit CPP in the α6L9′S mice suggests that it is rewarding for these mice. Interestingly, the CPP difference scores for the saline-injected WT and mutant controls also suggest that WT mice habituate to the stress of repeated confinement in the least preferred chamber (i.e., their CPP difference scores are positive), while α6L9′S mutant mice become sensitized to it (i.e., their CPP difference scores are negative). For the WT mice, the CPP dose-response data were fitted to the Michaelis–Menten equation (see Materials and Methods) with an ED_50_ value of 4 ± 3 ng/kg, whereas the lowest dose tested for the α6L9′S mice elicited a near maximal response, consistent with an ED_50_ value < 1 ng/kg (**Figures 4A,B**). This confirms that mice with hypersensitive α6L9′S^∗^ nAChRs were more sensitive to TC299423-induced CPP than WT mice. Thus, low doses of TC299423 are more rewarding for the hypersensitive α6L9’S mutant mice than the WT.

Even though TC299423 had no significant overall effect on CPP in the WT mice (one-way ANOVA, Tukey HSD, *p* = 0.23), a *t*-test revealed a marginally significant increase in CPP difference score for the 0.012 mg/kg TC299423-injected group, compared to the saline-injected control (*p* = 0.047) (**Figure 4A**). Using this dose, we examined the importance of individual nAChR subunits on TC299423 reward-related behavior. We compared WT mice to α6KO, α4KO, and β2KO mice, all injected with 0.012 mg/kg TC299423. Here, we observed a significant overall different response to TC299423 among the genotypes [one-way ANOVA, *F*_(3,47)_ = 4.825, *p* = 0.005] (**Figure 4C**). Genetic deletion of the α6 and β2 subunits dramatically reduced the CPP difference scores (*p* < 0.05), whereas deletion of the α4 subunit had no effect on the difference scores (**Figure 4C**). These data suggest that (1) a low dose of TC299423 (0.012 mg/kg) is weakly rewarding for WT mice, and (2) this rewarding effect is mediated by the activation of α6(non-α4)β2^∗^ nAChRs.

### Food Rewards or Nicotine IVSA

**FIGURE 5:**
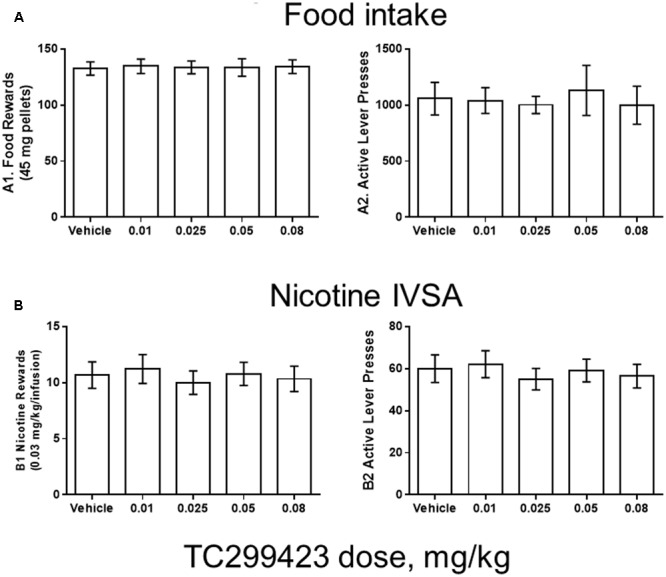
TC299423 does not alter nicotine IVSA. Pre-injection of TC299423 in rats at doses of 0.01, 0.025, 0.05, or 0.08 mg/kg did not affect food intake **(A)** or nicotine IVSA **(B)** compared to vehicle control (saline). (A1) Dose-response relation for earned food rewards (*n* = 8) (ns, *p* = 0.9967). (A2) Dose-response relation for active lever presses during food responding (ns, *p* = 0.7861). (B1) Dose-response relation for earned nicotine infusions (0.03 mg/kg) (*n* = 9) (ns, *p* = 0.6331). (B2) Dose-response relation for active lever presses during nicotine self-administration (ns, *p* = 0.5798). Data presented are mean ± SEM and analyzed using one-way repeated measures ANOVA.

The CPP data above suggest that low doses of TC299423 are weakly rewarding for WT mice but do not provide any information about the potential effects of TC299423 on the motivational properties of other substances, such as food and nicotine. Previous results show that nAChR partial agonists such as varenicline and (+)-PHT (**Figure 1**) block nicotine-induced IVSA and CPP (Rollema et al., 2007; Carroll et al., 2015). Thus, we tested the effects of TC299423 on male Wistar rats trained to press a lever for food pellets or intravenous nicotine infusions (**Figure 5**).

Rats were trained using 45 mg food pellets on a FR5TO20 schedule. Rats were then permitted to respond for food (*n* = 8) or nicotine infusions (0.03 mg/kg/inf) (*n* = 9) during 1 h daily sessions under the FR5TO20 schedule. TC299423 or vehicle was administered (*ip*, 1 mL/300 g weight, doses indicated in **Figure 5**) 20 min before placement in operant boxes. Compared to vehicle, rats pre-injected with doses of 0.01, 0.025, 0.05, or 0.08 mg/kg TC299423 showed no statistically significant changes in responding for food pellets or nicotine infusions [one-way ANOVA: Nicotine (*p* = 0.6331), Food (*p* = 0.9967)]. In neither case did TC299423 change the number of active lever presses. Thus, low-dose TC299423 did not inhibit the rewarding effects of food or nicotine.

### TC299423 Effect on Locomotion

**FIGURE 6:**
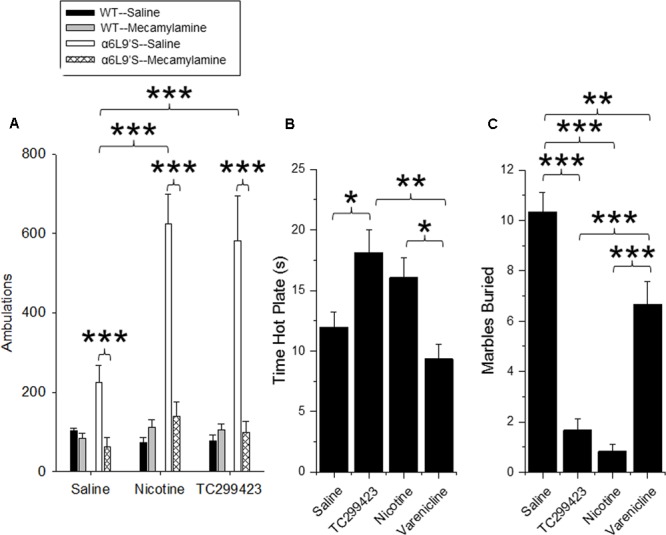
Locomotor, anxiolytic and analgesic effects of TC299423. **(A)** Locomotor activity of WT and α6L9′S mice in a novel environment. Mice were injected ip with saline, 0.08 mg/kg nicotine, or 0.09 mg/kg TC299423, and their locomotor activity was measured. Mice were also pre-injected with saline or 1 mg/kg mecamylamine prior to testing (*n* = 8–32). Pre-injection with mecamylamine blocked nicotine- and TC29942-mediated increase in locomotor activity in the α6L9’S mice. **(B)** The effects of nicotine, varenicline, and TC299423 (0.3 mg/kg for each) on antinociception were measured using the hot-plate test (*n* = 7–8 mice). **(C)** The effects of nicotine, varenicline, and TC299423 (0.3 mg/kg for each) on marble burying were measured (*n* = 8–11 mice). In all panels, data presented are mean ± SEM: ^∗^*p* < 0.05, ^∗∗^*p* < 0.01, ^∗∗∗^*p* < 0.001 [Two-way ANOVA with *post hoc* Tukey **(A)** or One-way ANOVA with *post hoc* Tukey **(B,C)**].

Locomotor responses to novel stimuli are an index of animal exploration and anxiety that can also provide a predictive factor for responses to rewarding drugs (Antoniou et al., 2008). Mice that exhibit greater motor activity (high responders) in a novel environment are more likely to be susceptible to rewarding properties of drugs, compared to mice that exhibit low activity (low responders). Thus, we used a locomotor assay to compare the *in vivo* effects of low doses of TC299423 and nicotine on WT and α6L9′S mice (see Materials and Methods, **Figure 6A**). Mice were injected *ip* with saline, nicotine (0.08 mg/kg), or TC299423 (0.09 mg/kg) and their ambulatory activity in a novel environment was recorded (**Figure 6A**). (Doses of 0.08 mg/kg nicotine and 0.09 mg/kg TC299423 are equimolar, 0.5 μmol/kg). To determine whether the effects of nicotine and TC299423 on ambulation were driven by nAChR activation, the mice were also pre-injected with either saline or mecamylamine (1 mg/kg) (**Figure 6A**). Both genotype and drug treatment significantly affected ambulation [two-way ANOVA: genotype, *F*_(1,72)_ = 15.98, *p <* 0.005; drug effect, *F*_(5,72)_ = 7.70, *p* < 0.005]. Nicotine and TC299423 at these doses dramatically increased the ambulatory activity of the mutant α6L9′S, but not WT, mice (**Figure 6A**). The increases were blocked by mecamylamine pre-injections. Neither TC299423 nor nicotine affected WT ambulation, and there were no significant differences between the ambulatory activity of WT and α6L9′S mice pre-injected mecamylamine. Interestingly, even the saline-injected α6L9′S mice showed significantly more ambulation than the WT (*p* < 0.01). This difference was also blocked by mecamylamine pre-injection (**Figure 6A**), suggesting that it is mediated by the endogenous activation of α6L9′S^∗^ nAChRs. Thus, consistent with the CPP results above and previous data (Drenan et al., 2008), the locomotor response of the mutant α6L9′S mice was more sensitive to nicotine and TC299423 than that of the WT mice. The locomotor data also confirm the activation of α6L9′S^∗^ nAChRs by a low dose of TC299423 (0.09 mg/kg in this case).

### TC299423 Effect on Antinociception

Previous data show that nicotine is antinociceptive and this effect is mediated by α4β2^∗^ (Damaj et al., 2007) and α6^∗^ nAChRs (Wieskopf et al., 2015). Because TC299423 is an agonist for α4β2^∗^ nAChRs at higher doses, we tested its antinociceptive properties at a dose of 0.3 mg/kg in WT mice using a hot plate assay, and compared its antinociceptive properties to those of 0.3 mg/kg nicotine and 0.3 mg/kg varenicline (**Figure 6B**). The drug treatments significantly affected responses of WT mice measured with the hot plate test [One-way ANOVA, *F*_(3,27)_ = 6.826, *p* = 0.0014]. The antinociception elicited by TC299423 was similar to that elicited by 0.3 mg/kg nicotine. The antinociceptive effects of both nicotine and TC299423 were significantly greater than that of varenicline (**Figure 6B**, *p* < 0.05).

### TC299423 Effect on Anxiety-Related Behavior

The anxiolytic effects of nicotine may contribute to its rewarding effects, and appear to be mediated by α4β2^∗^ nAChRs (Turner et al., 2010; Anderson and Brunzell, 2012). Marble burying behavior is a widely used (Deacon, 2006; Yohn and Blendy, 2017) measure of anxiety-related and compulsive behavior in mice, though interpretation may be complex (Thomas et al., 2009; Wolmarans de et al., 2016). To compare the anxiolytic properties of TC299423, varenicline, and nicotine, WT mice were administered saline, nicotine (0.3 mg/kg), varenicline (0.3 mg/kg), or TC299423 (0.3 mg/kg) and evaluated with the marble burying test (**Figure 6C**). Overall, the drugs significantly affected the number of marbles buried [One-way ANOVA, *F*_(3,33)_ = 45.65, *p* < 0.001]. Mice buried significantly fewer marbles following nicotine, TC299423, or varenicline administration than following saline administration. Further, mice buried fewer marbles after nicotine or TC299423 injections than varenicline injections (*p* < 0.001, **Figure 6C**). Note that neither nicotine, nor TC299423, significantly reduced locomotion in WT mice (**Figure 6A**), rendering it unlikely that the decrease in marble burying is caused by physical sedation.

## Discussion

Our results show that TC299423 is a potent and selective agonist for α6β2^∗^ and α4β2^∗^ nAChRs, compared to α3β4^∗^ nAChRs (**Figure 3** and **Tables 1, 2**). In addition, radioligand displacement assays indicate that TC299423 exhibits virtually no off target interactions (see Supplementary Table S3). TC299423 is orally available and crosses the blood-brain barrier (**Figure 2** and Supplementary Table S1). TC299423 has a longer half-life than nicotine (Petersen et al., 1984) and remains in the brain for at least 60 min following *ip* injection or oral administration. Experiments with synaptosomes show that TC299423 elicits α6^∗^ nAChR-mediated, striatal [^3^H]dopamine release. Consistent with these data, behavioral assays such as CPP (**Figure 4**) and locomotion in a novel environment (**Figure 6A**), which depend on dopamine release, suggest that TC299423 can elicit dopamine release *in vivo*. TC299423 is roughly as effective as nicotine in eliciting locomotor responses in α6L9′S mice (**Figure 6A**). Thus, TC299423 is a novel nAChR agonist that may be useful in studying of nAChR function and physiology in both *in vitro* and *in vivo* systems.

Our *in vitro* and *in vivo* data characterizing the properties of TC299423, along with those reported on the tropane compound, (+)-pyrido[3,4]homotropane [(+)-PHT] (Carroll et al., 2015) (see **Figure 1** for structure), are promising indications that selective α6β2^∗^ nAChR agonists can be identified. Given the pharmacological similarities between α6^∗^ and α4^∗^ nAChRs (Breining et al., 2009), it has been challenging to identify agonists that show even modest selectivity. Previous studies indicate that nicotine is more potent on α4(non-α6)β2^∗^-nAChRs than it is on α6(non-α4)β2^∗^ nAChRs, though it is even more potent on α4α6β2^∗^ nAChRs (Salminen et al., 2007; Walsh et al., 2008; Grady et al., 2010). TC299423, in contrast, appears to be more potent at α6(non-α4)β2^∗^ than at α4(non-α6)β2^∗^-nAChRs (see **Table 1** and **Figure 3**). Also, it is encouraging that the fold difference between the EC_50_ values for α6β2^∗^ vs. α4β2^∗^ nAChRs is consistent using two distinct assays for receptor function: patch-clamp assays on identified subtypes expressed in cultured cells and [^3^H]dopamine release assays using mouse synaptosomes (see **Table 1** and **Figure 3**). A similar pattern was observed for (+)-PHT (Carroll et al., 2015), but TC299423 is considerably more potent than (+)-PHT. An analog of TC299423, 2-(pyridine-3-yl)quinuclidine [see **Figure 1** and (Grady et al., 2010)], also exhibited a similar pattern in potency among α6β2^∗^ and α4β2^∗^ nAChRs. However, PHT was also relatively potent on α3β4 and α7 nAChRs (EC_50_ values of 0.43 and 0.66 μM, respectively). Thus, TC299423 is a notable advance compared to our previous series of small molecules (Breining et al., 2009; Grady et al., 2010). Although TC299423 shows a preference for α6^∗^ nAChRs, the difference in potency between α6^∗^ and α4^∗^ nAChRs is not sufficient to label it as an α6^∗^ selective drug. Thus, the identification of a truly selective small molecule for α6^∗^ nAChRs is still to come.

Our pharmacokinetic assays confirm that oral and *ip* administrations of TC299423 achieve brain concentrations that are sufficient for activating nAChRs. Hence, we investigated the rewarding properties of TC299423 using CPP and nicotine IVSA assays. Pharmacological and gene deletion studies show that the β2, α4, and α6 nAChR subunits are critical for nicotine-induced CPP (Walters et al., 2006; Sanjakdar et al., 2014). Transgenic mice expressing hypersensitive α6^∗^ nAChRs exhibit significant CPP in response to a range of TC299423 doses (see **Figure 4**). TC299423 doses in this range do not have a significant overall effect on CPP in WT mice. However, significant differences between the CPP of WT and nAChR KO mice (α6KO and β2KO) in response to a single TC299423 dose (0.012 μg/kg) suggest that: (1) it has a weakly rewarding effect at this dose in WT mice, and (2) α6 and β2 nAChR subunits (but not α4) mediate this effect (see **Figure 4A**). We did not observe any difference between TC299423-induced CPP in the WT and α4KO mice. Thus, the rewarding effects of TC299423 at this dose are likely to be mediated by α6(non-α4)β2^∗^ nAChRs, rather than α4α6β2^∗^ or α4β2 nAChRs. Our KO data also suggest that TC299423 reward-related behavior in WT and α6L9′S mice is mediated via nAChRs, rather than via off-target responses. The contribution of the α6^∗^ nAChRs to TC299423-induced CPP is reinforced by its potency in establishing CPP in the α6L9′S mice (see **Figure 4C**). The enhanced sensitivity of α6L9′S mice to TC299423-induced CPP is consistent with previous cellular and behavioral data showing an enhanced sensitivity to nicotine as well (Drenan et al., 2008; Drenan and Lester, 2012). Interestingly, the effects of TC299423 on CPP in α6KO and α4KO differ from that of nicotine (Sanjakdar et al., 2014). (+)-PHT also elicits CPP in mice, but at higher doses than TC299423 (Carroll et al., 2015).

We did not find any effects of low-dose TC299423 on nicotine IVSA or food reward in rats. Nicotine has reinforcing behavioral effects through activation and desensitization of nAChRs in the central nervous system. β2^∗^ nAChRs, which often contain α4 and/or α6 subunits, are necessary for nicotine IVSA and CPP (Picciotto et al., 1998; Maskos et al., 2005; Walters et al., 2006). α4^∗^ nAChRs are sufficient for establishing nicotine CPP (Tapper et al., 2004; McGranahan et al., 2011), but apparently not necessary (Cahir et al., 2011). They are also important, if not necessary, for nicotine IVSA (Cahir et al., 2011). Similarly, α6^∗^ nAChRs are sufficient for nicotine CPP (Drenan et al., 2012), but not necessary (Sanjakdar et al., 2014), and appear to be necessary for nicotine IVSA (Pons et al., 2008). Recent investigations using smoking-relevant concentrations of nicotine highlight the importance of activating VTA nAChRs that contain both the α4 and α6 nAChR subunits (Liu et al., 2012; Engle et al., 2013) and suggest that α4α6β2^∗^ nAChRs are a primary target for nicotine. In the context of nicotine IVSA, α4^∗^, α6^∗^, and β2^∗^ nAChRs are certainly involved, but one must also consider the involvement of α4α6β2^∗^ nAChRs.

While TC299423 had no effect on nicotine IVSA, several nAChR ligands have been shown to reduce nicotine IVSA: varenicline (Rollema et al., 2007), sazetidine-A (Pałczyñska et al., 2012), mecamylamine (DeNoble and Mele, 2006; Fowler and Kenny, 2014), bupropion (Bruijnzeel and Markou, 2003), and cytisine (Radchenko et al., 2015). Multiple mechanisms can account for the failure of a low-dose of TC299423 to suppress nicotine IVSA. First, the suppressive effects of nicotine, varenicline, and sazetidine-A may arise from their effects on α4β2^∗^, rather than α6β2, nAChRs. Second, the rewarding effects of TC299423 at the dosages used may be insufficient to substitute for those of nicotine. Third, we note a key difference between TC299423 and drugs that have been shown to alter nicotine reinforcement: varenicline, cytisine, and bupropion are all partial agonists shown to decrease nicotine IVSA or nicotine CPP (Bruijnzeel and Markou, 2003; Rollema et al., 2010) but they also act as nAChR antagonists (Slemmer et al., 2000; Mihalak et al., 2006; Papke et al., 2011). Additionally, previous studies show that potent nAChR antagonists decrease nicotine CPP and nicotine IVSA (DeNoble and Mele, 2006; Sanjakdar et al., 2014). We did not observe that TC299423 acts as an antagonist on nAChRs and this could be a key mechanistic reason why it does not alter nicotine reinforcement. Finally, TC299423 may enhance both the reinforcing and aversive properties of nicotine, effectively neutralizing the effects on nicotine IVSA. There is a dense population of α6^∗^ nAChRs in the medial habenula (Henderson et al., 2014; Shih et al., 2014) and this region regulates nicotine aversion (Fowler et al., 2011). However, all reports of this phenomena point to α2^∗^, α5^∗^, and β4^∗^ nAChRs mediating aversion to nicotine (Salas et al., 2009; Fowler et al., 2011); and direct involvement of α6 nAChR subunits has yet to be examined. If α6^∗^ nAChRs also mediate aversive responses in the medial habenula, TC299423 stimulation of α6^∗^ nAChRs may produce a simultaneous enhancement of rewarding and aversive stimuli. While it is currently unknown whether α6^∗^ nAChRs play a role in nicotine aversion, we note that α6^∗^ nAChRs play a critical role in affective nicotine withdrawal behavior (Jackson et al., 2009).

Nicotine’s anxiolytic and analgesic properties are also considered contributing factors to nicotine addiction. We measured anxiety-related behavior and nociception in mice using the marble burying and hot-plate assays, respectively. Nicotine’s anxiolytic and nociceptive properties are believed to be primarily mediated by α4β2^∗^ nAChRs (Bannon et al., 1998; Vincler and Eisenach, 2005; Turner et al., 2010; Zhang et al., 2012; Hone et al., 2013). Thus, we used a higher dose of TC299423 for these assays (0.3 mg/kg) than in the CPP assays, to ensure activation of α4β2^∗^ receptors. We found that TC299423 resembles nicotine in both its anxiolytic and antinociceptive properties, and is more potent and/or efficacious in each than varenicline (see **Figure 6**).

Overall, our results represent a thorough pharmacological investigation (*in vivo* and *in vitro*) of the novel nAChR agonist, TC299423. Our data suggest that TC299423 is a full agonist at α4β2^∗^ nAChRs and support a previous conclusion that TC299423 is a partial agonist at α6β2^∗^ nAChRs (Post et al., 2015). Similar to CNS penetrant nAChR partial agonists (e.g., varenicline), TC299423 potently activates β2^∗^ nAChRs and exhibits suitable pharmacokinetic characteristics for use *in vivo.* TC299423 potently elicits reward-related behavior in hypersensitive α6L9′S mice and perhaps WT mice. At low doses, TC299423-initiated reward is primarily mediated through α6^∗^ nAChRs, not α4^∗^ nAChRs. Our studies of TC299423 and nicotine IVSA show that TC299423 at the low doses we tested is not efficacious in altering nicotine reinforcement. Nevertheless, TC299423 is a potent and novel nAChR agonist that could be useful for the future study of nAChR-related function and physiology.

## Ethics Statement

This study was performed with the consent of the Institutional Animal Care and Use Committees of the California Institute of Technology, the University of Colorado at Boulder, and the Mount Sinai School of Medicine.

## Author Contributions

Experiments performed by TW, BH, GV, CW, PD, BC, SG, and MM. Analysis by TW, BH, GV, CW, BC, SG, MM, DY, MB, and HL. Research direction by MM, DY, PK, MB, and HL. Manuscript preparation and revision by TW, BH, BC, SG, MM, DY, PK, MB, and HL. Funding obtained by BH, MM, PK, MB, and HL.

## Conflict of Interest Statement

When the research was conducted, MB and DY were employed by Targacept Inc. Targacept has since merged with Catalyst Biosciences. No entity or person now has any intellectual property, or commercial, or financial interest in TC299423. The other authors declare that the research was conducted in the absence of any commercial or financial relationships that could be construed as a potential conflict of interest.

## References

[B1] Anderson S. M. Brunzell D. H. (2012). Low dose nicotine and antagonism of β2 subunit containing nicotinic acetylcholine receptors have similar effects on affective behavior in mice. *PLOS ONE* 7:e48665 10.1371/journal.pone.0048665 PMC3492489 23144922

[B2] Antoniou K. Papathanasiou G. Papalexi E. Hyphantis T. Nomikos G. G. Spyraki C. (2008). Individual responses to novelty are associated with differences in behavioral and neurochemical profiles. *Behav. Brain Res.* 187 462–472. 10.1016/j.bbr.2007.10.010 18036673

[B3] Azam L. Maskos U. Changeux J. P. Dowell C. D. Christensen S. De Biasi M. (2010). α-Conotoxin BuIA[T5A;P6O]: a novel ligand that discriminates between α6β4 and α6β2 nicotinic acetylcholine receptors and blocks nicotine-stimulated norepinephrine release. *FASEB J.* 24 5113–5123. 10.1096/fj.10-166272 20739611 PMC3229426

[B4] Baddick C. G. Marks M. J. (2011). An autoradiographic survey of mouse brain nicotinic acetylcholine receptors defined by null mutants. *Biochem. Pharmacol.* 82 828–841. 10.1016/j.bcp.2011.04.019 21575611 PMC3162045

[B5] Bannon A. W. Decker M. W. Holladay M. W. Curzon P. Donnelly-Roberts D. Puttfarcken P. S. (1998). Broad-spectrum, non-opioid analgesic activity by selective modulation of neuronal nicotinic acetylcholine receptors. *Science* 279 77–81. 10.1126/science.279.5347.77 9417028

[B6] Breining S. R. Bencherif M. Grady S. R. Whiteaker P. Marks M. J. Wageman C. R. (2009). Evaluation of structurally diverse neuronal nicotinic receptor ligands for selectivity at the α6^∗^ subtype. *Bioorg. Med. Chem. Lett.* 19 4359–4363. 10.1016/j.bmcl.2009.05.085 19560354 PMC6107347

[B7] Breining S. R. Melvin M. Bhatti B. S. Byrd G. D. Kiser M. N. Hepler C. D. (2012). Structure-activity studies of 7-heteroaryl-3-azabicyclo[3.3.1]non-6-enes: a novel class of highly potent nicotinic receptor ligands. *J. Med. Chem.* 55 9929–9945. 10.1021/jm3011299 23025891

[B8] Bruijnzeel A. W. Markou A. (2003). Characterization of the effects of bupropion on the reinforcing properties of nicotine and food in rats. *Synapse* 50 20–28. 10.1002/syn.10242 12872290

[B9] Cahir E. Pillidge K. Drago J. Lawrence A. J. (2011). The necessity of α4^∗^ nicotinic receptors in nicotine-driven behaviors: dissociation between reinforcing and motor effects of nicotine. *Neuropsychopharmacology* 36 1505–1517. 10.1038/npp.2011.35 21430644 PMC3096818

[B10] Carroll F. I. Navarro H. A. Mascarella S. W. Castro A. Luetje C. W. Wageman C. R. (2015). *In vitro* and *in vivo* neuronal nicotinic receptor properties of (+)- and (-)- pyrido[3,4] homotropane [(+)- and (-)-PHT]. (+)-PHT is a potent and selective full agonist at α6β2 containing neuronal nicotinic acetylcholine receptors. *ACS Chem. Neurosci.* 6 920–926. 10.1021/acschemneuro.5b00077 25891987 PMC5589077

[B11] Champtiaux N. Gotti C. Cordero-Erausquin M. David D. J. Przybylski C. Lena C. (2003). Subunit composition of functional nicotinic receptors in dopaminergic neurons investigated with knock-out mice. *J. Neurosci.* 23 7820–7829. 12944511 10.1523/JNEUROSCI.23-21-07820.2003 PMC6740613

[B12] Champtiaux N. Han Z. Y. Bessis A. Rossi F. M. Zoli M. Marubio L. (2002). Distribution and pharmacology of a6-containing nicotinic acetylcholine receptors analyzed with mutant mice. *J. Neurosci.* 22 1208–1217. 11850448 10.1523/JNEUROSCI.22-04-01208.2002 PMC6757563

[B13] Damaj M. I. Fonck C. Marks M. J. Deshpande P. Labarca C. Lester H. A. (2007). Genetic approaches identify differential roles for α4β2^∗^ nicotinic receptors in acute models of antinociception in mice. *J. Pharmacol. Exp. Ther.* 321 1161–1169. 10.1124/jpet.106.112649 17371806

[B14] Dani J. A. Balfour D. J. (2011). Historical and current perspective on tobacco use and nicotine addiction. *Trends Neurosci.* 34 383–392. 10.1016/j.tins.2011.05.001 21696833 PMC3193858

[B15] Deacon R. M. (2006). Digging and marble burying in mice: simple methods for in vivo identification of biological impacts. *Nat. Protoc.* 1 122–124. 10.1038/nprot.2006.20 17406223

[B16] DeNoble V. J. Mele P. C. (2006). Intravenous nicotine self-administration in rats: effects of mecamylamine, hexamethonium and naloxone. *Psychopharmacology (Berl)* 184 266–272. 10.1007/s00213-005-0054-z 16088413

[B17] Drenan R. M. Engle S. Lester H. A. McIntosh J. M. Brunzell D. H. (2012). *Activation of α6β2^∗^ Nicotinic Acetylcholine Receptors is Sufficient for Nicotine Reward. Program No. 455.03. Neuroscience Meeting Planner*. New Orleans, LA: Society for Neuroscience.

[B18] Drenan R. M. Grady S. R. Whiteaker P. McClure-Begley T. McKinney S. R. Miwa J. (2008). *In Vivo* activation of midbrain dopamine neurons via sensitized, high-affinity α6^∗^ nicotinic acetylcholine receptors. *Neuron* 60 123–136. 10.1016/j.neuron.2008.09.009 18940593 PMC2632732

[B19] Drenan R. M. Lester H. A. (2012). Insights into the neurobiology of the nicotinic cholinergic system and nicotine addiction from mice expressing nicotinic receptors harboring gain-of-function mutations. *Pharmacol. Rev.* 64 869–879. 10.1124/pr.111.004671 22885704 PMC3462994

[B20] Engle S. E. Shih P. Y. McIntosh J. M. Drenan R. M. (2013). alpha4alpha6beta2^∗^ nicotinic acetylcholine receptor activation on ventral tegmental area dopamine neurons is sufficient to stimulate a depolarizing conductance and enhance surface AMPA receptor function. *Mol. Pharmacol.* 84 393–406. 10.1124/mol.113.087346 23788655 PMC3876818

[B21] Fowler C. D. Kenny P. J. (2014). Nicotine aversion: neurobiological mechanisms and relevance to tobacco dependence vulnerability. *Neuropharmacology* 76 533–544. 10.1016/j.neuropharm.2013.09.008 24055497 PMC3858456

[B22] Fowler C. D. Lu Q. Johnson P. M. Marks M. J. Kenny P. J. (2011). Habenular α5 nicotinic receptor subunit signalling controls nicotine intake. *Nature* 471 597–601. 10.1038/nature09797 21278726 PMC3079537

[B23] Gotti C. Zoli M. Clementi F. (2006). Brain nicotinic acetylcholine receptors: native subtypes and their relevance. *Trends Pharmacol. Sci.* 27 482–491. 10.1016/j.tips.2006.07.004 16876883

[B24] Grady S. R. Drenan R. M. Breining S. R. Yohannes D. Wageman C. R. Fedorov N. B. (2010). Structural differences determine the relative selectivity of nicotinic compounds for native α4β2^∗^-, α6β2^∗^-, α3β4^∗^-, and α7 nicotinic acetylcholine receptors. *Neuropharmacology* 58 1054–1066. 10.1016/j.neuropharm.2010.01.013 20114055 PMC2849849

[B25] Grady S. R. Meinerz N. M. Cao J. Reynolds A. M. Picciotto M. R. Changeux J. P. (2001). Nicotinic agonists stimulate acetylcholine release from mouse interpeduncular nucleus: a function mediated by a different nAChR than dopamine release from striatum. *J. Neurochem.* 76 258–268. 10.1046/j.1471-4159.2001.00019.x 11145999

[B26] Grady S. R. Wageman C. R. Patzlaff N. E. Marks M. J. (2012). Low concentrations of nicotine differentially desensitize nicotinic acetylcholine receptors that include alpha5 or alpha6 subunits and that mediate synaptosomal neurotransmitter release. *Neuropharmacology* 62 1935–1943. 10.1016/j.neuropharm.2011.12.026 22239849 PMC3278500

[B27] Henderson B. Lester H. A. (2015). Inside-out neuropharmacology of nicotinic drugs. *Neuropharmacology* 96 178–193. 10.1016/j.neuropharm.2015.01.022 25660637 PMC4486611

[B28] Henderson B. J. Srinivasan R. Nichols W. A. Dilworth C. N. Gutierrez D. F. Mackey E. D. (2014). Nicotine exploits a COPI-mediated process for chaperone-mediated up-regulation of its receptors. *J. Gen. Physiol.* 143 51–66. 10.1085/jgp.201311102 24378908 PMC3874574

[B29] Hone A. J. Ruiz M. Scadden M. Christensen S. Gajewiak J. Azam L. (2013). Positional scanning mutagenesis of α-conotoxin PeIA identifies critical residues that confer potency and selectivity for α6/α3β2β3 and α3β2 nicotinic acetylcholine receptors. *J. Biol. Chem.* 288 25428–25439. 10.1074/jbc.M113.482059 23846688 PMC3757205

[B30] Hone A. J. Scadden M. Gajewiak J. Christensen S. Lindstrom J. McIntosh J. M. (2012). α-Conotoxin PeIA[S9H,V10A,E14N] potently and selectively blocks α6β2β3 versus α6β4 nicotinic acetylcholine receptors. *Mol. Pharmacol.* 82 972–982. 10.1124/mol.112.080853 22914547 PMC3477225

[B31] Hussmann G. P. Kellar K. J. (2012). A new radioligand binding assay to measure the concentration of drugs in rodent brain ex vivo. *J. Pharmacol. Exp. Ther.* 343 434–440. 10.1124/jpet.112.198069 22899751 PMC3477219

[B32] Jackson K. J. McIntosh J. M. Brunzell D. H. Sanjakdar S. S. Damaj M. I. (2009). The role of alpha6-containing nicotinic acetylcholine receptors in nicotine reward and withdrawal. *J. Pharmacol. Exp. Ther.* 331 547–554. 10.1124/jpet.109.155457 19644040 PMC2775251

[B33] Janowsky D. S. el-Yousef M. K. Davis J. M. Sekerke H. J. (1972). A cholinergic-adrenergic hypothesis of mania and depression. *Lancet* 2 632–635. 10.1016/S0140-6736(72)93021-8 4116781

[B34] Jensen A. A. Frolund B. Liljefors T. Krogsgaard-Larsen P. (2005). Neuronal nicotinic acetylcholine receptors: structural revelations, target identifications, and therapeutic inspirations. *J. Med. Chem.* 48 4705–4745. 10.1021/jm040219e 16033252

[B35] Kenny P. J. Chartoff E. Roberto M. Carlezon W. A. Jr. Markou A. (2008). NMDA Receptors regulate nicotine-enhanced brain reward function and intravenous nicotine self-administration: role of the ventral tegmental area and central nucleus of the amygdala. *Neuropsychopharmacology* 34 266–281. 10.1038/npp.2008.58 18418357 PMC2654386

[B36] Kuryatov A. Lindstrom J. (2011). Expression of functional human α6β2β3 acetylcholine receptors in *Xenopus laevis* oocytes achieved through subunit chimeras and concatamers. *Mol. Pharmacol.* 79 126–140. 10.1124/mol.110.066159 20923852 PMC3014284

[B37] Kuryatov A. Luo J. Cooper J. Lindstrom J. (2005). Nicotine acts as a pharmacological chaperone to up-regulate human a4b2 acetylcholine receptors. *Mol. Pharmacol.* 68 1839–1851. 16183856 10.1124/mol.105.012419

[B38] Limapichat W. Dougherty D. A. Lester H. A. (2014). Subtype-specific mechanisms for functional interaction between α6β4^∗^ nicotinic acetylcholine receptors and P2X receptors. *Mol. Pharmacol.* 86 263–274. 10.1124/mol.114.093179 24966348 PMC4152909

[B39] Liu L. Zhao-Shea R. McIntosh J. M. Gardner P. D. Tapper A. R. (2012). Nicotine persistently activates ventral tegmental area dopaminergic neurons via nicotinic acetylcholine receptors containing alpha4 and alpha6 subunits. *Mol. Pharmacol.* 81 541–548. 10.1124/mol.111.076661 22222765 PMC3310415

[B40] Lowe J. A. III DeNinno S. L. Coe J. W. Zhang L. Mente S. Hurst R. S. (2010). A novel series of [3.2.1] azabicyclic biaryl ethers as α3β4 and α6/4β4 nicotinic receptor agonists. *Bioorg. Med. Chem. Lett.* 20 4749–4752. 10.1016/j.bmcl.2010.06.142 20663668

[B41] Mackey E. D. Engle S. E. Kim M. R. O’Neill H. C. Wageman C. R. Patzlaff N. E. (2012). α6^∗^ nicotinic acetylcholine receptor expression and function in a visual salience circuit. *J. Neurosci.* 32 10226–10237. 10.1523/JNEUROSCI.0007-12.2012 22836257 PMC3432940

[B42] Marks M. Grady S. Salminen O. Paley M. Wageman C. McIntosh J. (2014). α6β2^∗^-subtype nicotinic acetylcholine receptors are more sensitive than α4β2^∗^-subtype receptors to regulation by chronic nicotine administration. *J. Neurochem.* 130 185–198. 10.1111/jnc.12721 24661093 PMC4107044

[B43] Marks M. J. Meinerz N. M. Drago J. Collins A. C. (2007). Gene targeting demonstrates that α4 nicotinic acetylcholine receptor subunits contribute to expression of diverse [3H]epibatidine binding sites and components of biphasic 86Rb+ efflux with high and low sensitivity to stimulation by acetylcholine. *Neuropharmacology* 53 390–405. 10.1016/j.neuropharm.2007.05.021 17631923 PMC2577786

[B44] Marks M. J. Smith K. W. Collins A. C. (1998). Differential agonist inhibition identifies multiple epibatidine binding sites in mouse brain. *J. Pharmacol. Exp. Ther.* 285 377–386. 9536034

[B45] Marks M. J. Wageman C. R. Grady S. R. Gopalakrishnan M. Briggs C. A. (2009). Selectivity of ABT-089 for α4β2^∗^ and α6β2^∗^ nicotinic acetylcholine receptors in brain. *Biochem. Pharmacol.* 78 795–802. 10.1016/j.bcp.2009.05.022 19481067 PMC2772152

[B46] Marks M. J. Whiteaker P. Calcaterra J. Stitzel J. A. Bullock A. E. Grady S. R. (1999). Two pharmacologically distinct components of nicotinic receptor-mediated rubidium efflux in mouse brain require the b2 subunit. *J. Pharmacol. Exp. Ther.* 289 1090–1103. 10215692

[B47] Marks M. J. Whiteaker P. Collins A. C. (2006). Deletion of the a7, b2, or b4 nicotinic receptor subunit genes identifies highly expressed subtypes with relatively low affinity for [3H]epibatidine. *Mol. Pharmacol.* 70 947–959. 10.1124/mol.106.025338 16728647

[B48] Marubio L. M. del Mar Arroyo-Jimenez M. Cordero-Erausquin M. Lena C. Le Novere N. de Kerchove d’Exaerde A. (1999). Reduced antinociception in mice lacking neuronal nicotinic receptor subunits. *Nature* 398 805–810. 10.1038/19756 10235262

[B49] Maskos U. Molles B. E. Pons S. Besson M. Guiard B. P. Guilloux J. P. (2005). Nicotine reinforcement and cognition restored by targeted expression of nicotinic receptors. *Nature* 436 103–107. 10.1038/nature03694 16001069

[B50] Matta S. G. Balfour D. J. Benowitz N. L. Boyd R. T. Buccafusco J. J. Caggiula A. R. (2007). Guidelines on nicotine dose selection for *in vivo* research. *Psychopharm* 190 269–319. 10.1007/s00213-006-0441-0 16896961

[B51] McClure-Begley T. D. Wageman C. R. Grady S. R. Marks M. J. McIntosh J. M. Collins A. C. (2012). A novel α-conotoxin MII-sensitive nicotinic acetylcholine receptor modulates [3H]-GABA release in the superficial layers of the mouse superior colliculus. *J. Neurochem.* 122 48–57. 10.1111/j.1471-4159.2012.07759.x 22506481 PMC4026281

[B52] McGranahan T. M. Patzlaff N. E. Grady S. R. Heinemann S. F. Booker T. K. (2011). α4β2 nicotinic acetylcholine receptors on dopaminergic neurons mediate nicotine reward and anxiety relief. *J. Neurosci.* 31 10891–10902. 10.1523/JNEUROSCI.0937-11.2011 21795541 PMC3539812

[B53] Mihalak K. B. Carroll F. I. Luetje C. W. (2006). Varenicline is a partial agonist at α4β2 and a full agonist at α7 neuronal nicotinic receptors. *Mol. Pharmacol.* 70 801–805. 10.1124/mol.106.025130 16766716

[B54] Nelson M. E. Kuryatov A. Choi C. H. Zhou Y. Lindstrom J. (2003). Alternate stoichiometries of a4b2 nicotinic acetylcholine receptors. *Mol. Pharmacol.* 63 332–341. 10.1124/mol.63.2.332 12527804

[B55] Newhouse P. Kellar K. Aisen P. White H. Wesnes K. Coderre E. (2012). Nicotine treatment of mild cognitive impairment: a 6-month double-blind pilot clinical trial. *Neurology* 78 91–101. 10.1212/WNL.0b013e31823efcbb 22232050 PMC3466669

[B56] Pałczyñska M. M. Jindrichova M. Gibb A. J. Millar N. S. (2012). Activation of α7 nicotinic receptors by orthosteric and allosteric agonists: influence on single-channel kinetics and conductance. *Mol. Pharmacol.* 82 910–917. 10.1124/mol.112.080259 22874415 PMC3477227

[B57] Papke R. L. Trocme-Thibierge C. Guendisch D. Al Rubaiy S. A. Bloom S. A. (2011). Electrophysiological perspectives on the therapeutic use of nicotinic acetylcholine receptor partial agonists. *J. Pharmacol. Exp. Ther.* 337 367–379. 10.1124/jpet.110.177485 21285282 PMC3083103

[B58] Perez-Alvarez A. Hernandez-Vivanco A. McIntosh J. M. Albillos A. (2011). Native α6β4^∗^ nicotinic receptors control exocytosis in human chromaffin cells of the adrenal gland. *FASEB J.* 26 346–354. 10.1096/fj.11-190223 21917987 PMC3250250

[B59] Petersen D. R. Norris K. J. Thompson J. A. (1984). A comparative study of the disposition of nicotine and its metabolites in three inbred strains of mice. *Drug Metab. Dispos.* 12 725–731. 6150822

[B60] Picciotto M. R. Addy N. A. Mineur Y. S. Brunzell D. H. (2008). It is not “either/or”: Activation and desensitization of nicotinic acetylcholine receptors both contribute to behaviors related to nicotine addiction and mood. *Prog. Neurobiol.* 84 329–342. 10.1016/j.pneurobio.2007.12.005 18242816 PMC2390914

[B61] Picciotto M. R. Zoli M. Rimondini R. Lena C. Marubio L. M. Pich E. M. (1998). Acetylcholine receptors containing the b2 subunit are involved in the reinforcing properties of nicotine. *Nature* 391 173–177. 10.1038/34413 9428762

[B62] Pons S. Fattore L. Cossu G. Tolu S. Porcu E. McIntosh J. M. (2008). Crucial role of α4 and α6 nicotinic acetylcholine receptor subunits from ventral tegmental area in systemic nicotine self-administration. *J. Neurosci.* 28 12318–12327. 10.1523/JNEUROSCI.3918-08.2008 19020025 PMC2819191

[B63] Post M. Lester H. A. Dougherty D. A. (2017). Probing for and quantifying agonist hydrogen bonds in α6β2 nicotinic acetylcholine receptors. *Biochemistry* 56 1836–1840. 10.1021/acs.biochem.7b00213 28287260 PMC6075822

[B64] Post M. R. Limapichat W. Lester H. A. Dougherty D. A. (2015). Heterologous expression and nonsense suppression provide insights into agonist behavior at α6β2 nicotinic acetylcholine receptors. *Neuropharmacology* 97 376–382. 10.1016/j.neuropharm.2015.04.009 25908401 PMC4635625

[B65] Quik M. Wonnacott S. (2011). α6β2^∗^ and α4β2^∗^ nicotinic acetylcholine receptors as drug targets for Parkinson’s disease. *Pharmacol. Rev.* 63 938–966. 10.1124/pr.110.003269 21969327 PMC3186078

[B66] Radchenko E. V. Dravolina O. A. Bespalov A. Y. (2015). Agonist and antagonist effects of cytisine in vivo. *Neuropharmacology* 95 206–214. 10.1016/j.neuropharm.2015.03.019 25839895

[B67] Rollema H. Chambers L. K. Coe J. W. Glowa J. Hurst R. S. Lebel L. A. (2007). Pharmacological profile of the α4β2 nicotinic acetylcholine receptor partial agonist varenicline, an effective smoking cessation aid. *Neuropharmacology* 52 985–994. 10.1016/j.neuropharm.2006.10.016 17157884

[B68] Rollema H. Shrikhande A. Ward K. M. Tingley F. D. III Coe J. W. O’Neill B. T. (2010). Pre-clinical properties of the α4β2 nicotinic acetylcholine receptor partial agonists varenicline, cytisine and dianicline translate to clinical efficacy for nicotine dependence. *Br. J. Pharmacol.* 160 334–345. 10.1111/j.1476-5381.2010.00682.x 20331614 PMC2874855

[B69] Salas R. Sturm R. Boulter J. De Biasi M. (2009). Nicotinic receptors in the habenulo-interpeduncular system are necessary for nicotine withdrawal in mice. *J. Neurosci.* 29 3014–3018. 10.1523/JNEUROSCI.4934-08.2009 19279237 PMC3862238

[B70] Salminen O. Drapeau J. A. McIntosh J. M. Collins A. C. Marks M. J. Grady S. R. (2007). Pharmacology of a-Conotoxin MII-sensitive subtypes of nicotinic acetylcholine receptors isolated by breeding of null mutant mice. *Mol. Pharmacol.* 71 1563–1571. 10.1124/mol.106.031492 17341654

[B71] Salminen O. Murphy K. L. McIntosh J. M. Drago J. Marks M. J. Collins A. C. (2004). Subunit composition and pharmacology of two classes of striatal presynaptic nicotinic acetylcholine receptors mediating dopamine release in mice. *Mol. Pharmacol.* 65 1526–1535. 10.1124/mol.65.6.1526 15155845

[B72] Sanjakdar S. S. Maldoon P. P. Marks M. J. Brunzell D. H. Maskos U. McIntosh J. M. (2014). Differential Roles of α6β2^∗^ and α4β2^∗^ neuronal nicotinic receptors in nicotine- and cocaine-conditioned reward in mice. *Neuropsychopharmacology* 40 350–360. 10.1038/npp.2014.177 25035086 PMC4443947

[B73] Shih P. Y. Engle S. E. Oh G. Deshpande P. Puskar N. L. Lester H. A. (2014). Differential expression and function of nicotinic acetylcholine receptors in subdivisions of medial habenula. *J. Neurosci.* 34 9789–9802. 10.1523/jneurosci.0476-14.2014 25031416 PMC4099552

[B74] Slemmer J. E. Martin B. R. Damaj M. I. (2000). Bupropion is a nicotinic antagonist. *J. Pharmacol. Exp. Ther.* 295 321–327. 10991997

[B75] Srinivasan R. Henderson B. J. Lester H. A. Richards C. I. (2014). Pharmacological chaperoning of nAChRs: a therapeutic target for Parkinson’s disease. *Pharmacol. Res.* 83 20–29. 10.1016/j.phrs.2014.02.005 24593907 PMC6075820

[B76] Srinivasan R. Pantoja R. Moss F. J. Mackey E. D. W. Son C. Miwa J. (2011). Nicotine upregulates α4β2 nicotinic receptors and ER exit sites via stoichiometry-dependent chaperoning. *J. Gen. Physiol.* 137 59–79. 10.1085/jgp.201010532 21187334 PMC3010053

[B77] Strachan J. P. Kombo D. C. Mazurov A. Heemstra R. Bhatti B. S. Akireddy R. (2014). Identification and pharmacological characterization of 3,6-diazabicyclo[3.1.1]heptane-3-carboxamides as novel ligands for the α4β2 and α6/α3β2β3 nicotinic acetylcholine receptors (nAChRs). *Eur. J. Med. Chem.* 86 60–74. 10.1016/j.ejmech.2014.08.019 25147147

[B78] Tapper A. R. McKinney S. L. Nashmi R. Schwarz J. Deshpande P. Labarca C. (2004). Nicotine activation of α4^∗^ receptors: sufficient for reward, tolerance and sensitization. *Science* 306 1029–1032. 10.1126/science.1099420 15528443

[B79] Thomas A. Burant A. Bui N. Graham D. Yuva-Paylor L. A. Paylor R. (2009). Marble burying reflects a repetitive and perseverative behavior more than novelty-induced anxiety. *Psychopharmacology* 204 361–373. 10.1007/s00213-009-1466-y 19189082 PMC2899706

[B80] Turner J. R. Castellano L. M. Blendy J. A. (2010). Nicotinic partial agonists varenicline and sazetidine-A have differential effects on affective behavior. *J. Pharmacol. Exp. Ther.* 334 665–672. 10.1124/jpet.110.166280 20435920 PMC2913767

[B81] Vincler M. A. Eisenach J. C. (2005). Knock down of the α5 nicotinic acetylcholine receptor in spinal nerve-ligated rats alleviates mechanical allodynia. *Pharmacol. Biochem. Behav.* 80 135–143. 10.1016/j.pbb.2004.10.011 15652389

[B82] Walsh H. Govind A. P. Mastro R. Hoda J. C. Bertrand D. Vallejo Y. (2008). Upregulation of nicotinic receptors by nicotine varies with receptor subtype. *J. Biol. Chem.* 283 6022–6032. 10.1074/jbc.M703432200 18174175

[B83] Walters C. L. Brown S. Changeux J. P. Martin B. Damaj M. I. (2006). The b2 but not a7 subunit of the nicotinic acetylcholine receptor is required for nicotine-conditioned place preference in mice. *Psychopharmacology (Berl)* 184 339–344. 10.1007/s00213-005-0295-x 16416156

[B84] Wieskopf J. S. Mathur J. Limapichat W. Post M. R. Al-Qazzaz M. Sorge R. E. (2015). The nicotinic α6 subunit gene determines variability in chronic pain sensitivity via cross-inhibition of P2X2/3 receptors. *Sci. Transl. Med.* 7 287ra272 10.1126/scitranslmed.3009986 PMC5018401 25972004

[B85] Wolmarans de W. Stein D. J. Harvey B. H. (2016). Of mice and marbles: novel perspectives on burying behavior as a screening test for psychiatric illness. *Cogn. Affect. Behav. Neurosci.* 16 551–560. 10.3758/s13415-016-0413-8 26920212

[B86] Xiao C. Srinivasan R. Drenan R. M. Mackey E. D. McIntosh J. M. Lester H. A. (2011). Characterizing functional α6β2 nicotinic acetylcholine receptors in vitro: mutant β2 subunits improve membrane expression, and fluorescent proteins reveal responsive cells. *Biochem. Pharmacol.* 82 852–861. 10.1016/j.bcp.2011.05.005 21609715 PMC3162078

[B87] Yohn N. L. Blendy J. A. (2017). Adolescent chronic unpredictable stress exposure is a sensitive window for long-term changes in adult behavior in mice. *Neuropsychopharmacology* 42 1670–1678. 10.1038/npp.2017.11 28094283 PMC5518894

[B88] Zhang J. Xiao Y.-D. Jordan K. G. Hammond P. S. Van Dyke K. M. Mazurov A. A. (2012). Analgesic effects mediated by neuronal nicotinic acetylcholine receptor agonists: Correlation with desensitization of α4β2^∗^ receptors. *Eur. J. Pharm. Sci.* 47 813–823. 10.1016/j.ejps.2012.09.014 23036283

[B89] Zhang Z. Zheng G. Pivavarchyk M. Deaciuc A. G. Dwoskin L. P. Crooks P. A. (2011). Novel bis-, tris-, and tetrakis-tertiary amino analogs as antagonists at neuronal nicotinic receptors that mediate nicotine-evoked dopamine release. *Bioorg. Med. Chem. Lett.* 21 88–91. 10.1016/j.bmcl.2010.11.070 21147530 PMC3725996

