# Supplementary material for: TC299423, a Novel Agonist for Nicotinic Acetylcholine Receptors
Source: Front Pharmacol. 2017 Sep 26;8:641. doi: 10.3389/fphar.2017.00641 (PMC5626944; doi:10.3389/fphar.2017.00641)
Supplement: Supplementary file 1 [file Presentation_1.pdf]

TC299423, A Novel Partial Agonist for Nicotinic Acetylcholine Receptors

Teagan R. Wall[1¶], Brandon J. Henderson[2¶], George Voren[3], Charles R. Wageman[4], Purnima Deshpande[1], Bruce N. Cohen[1], Sharon R. Grady[4], Michael J. Marks[4, 5], Daniel Yohannes[6], Paul J. Kenny[3], Merouane Bencherif[6], Henry A. Lester[1*]

Supplementary information

S1 Table. Brain and Plasma concentrations of TC299423 measured by [^{125}I]epibatidine displacement

Brain measurements

Time, min	***K_i, pM***	***[TC299423] in assay, nM***	***[TC299423] in brain, pmol/mg[a]***
Control	23 ± 2.3	0	0
5	27 ± 2.5	2.2 ± 1.1	45 ± 25
10	31 ± 1.7	4.4 ± 0.9	88 ± 18
20	29 ± 0.4	3.7 ± 0.2	73 ± 4.0

Blood measurements

Time, min	***Vol serum for IC_{50}, µL***	***[TC299423] nM***
5	42 ± 9.7	250 ± 50
10	28 ± 5.5	370 ± 70
20	89 ± 18	120 ± 30

Data are expressed as mean ± SEM

[a], Data are pmol TC299423 / mg of brain tissue

**S2 Table. *In vitro* metabolism of TC299423, compared with varenicline.**										
	**Human microsomes**		**Rat microsomes**		**Turnover by human cytochrome P450 isoforms**					
	Clearance[a]	$T_{1/2}$[b]	Clearance[a]	$T_{1/2}$[b]	CYP1A2[c]	CYP2C9[c]	CYP2C19[c]	CYP2D6[c]	CYP3A4[c]	Human FMO3[c]
Varenicline	39	43	233	10	7	0	0	39	50	
TC299423	5	285	18	79	3	8	4	42	7	17

The assay mixtures (final incubation volume of 125 µL) in 0.255 M phosphate buffer with 0.575% (w/v) KCl (pH 7.4) contained PiB (1.0 µM), 2.0 mM nicotinamide-adenine dinucleotide phosphate (reduced form) (NADPH), and a recombinantly expressed cytochrome P450 enzyme preparation (1A2, 2C9, 2C19, 2D6, 3A4, and FMO3) at 100 pmol/mL. After a 10 min preincubation at 37 °C, the reaction was initiated by addition of 2.0 mM NADPH and incubated for 60 min. The reactions were terminated by the addition of two volumes of DMSO. Samples were subsequently centrifuged at 4,000 rpm for 10 min. From the resulting supernatant, 10 µL was analyzed by liquid chromatography/tandem mass spectrometry (LC-MS/MS). Control experiments were performed by substituting the active enzyme preparation by cell preparations containing no recombinant human CYP.

[a], Data for clearance are given as µL/min/mg protein.

[b], Unit for $T_{1/2}$ is min.

[c], Data for cytochrome P450 turnover and for human flavin-containing monooxygenase 3 (FMO3) are given as percent decline over 60 min.

S3 Table. Competition binding on 70 target proteins tested at a concentration of 1 µM TC299423			
**Inhibition**	**Assay Name**	**Radioligand/Substrate**	**Hit**
**12.86%**	Adenosine Transporter (h)	[3H]-NBTI	FALSE
**7.85%**	Adenosine, A1	[3H]CPX	FALSE
**1.51%**	Adenosine, A2A (h)	[3H]CGS 21680	FALSE
**-0.05%**	Adrenergic, Alpha 1A	[3H]-7-MeOxy-Prazosin	FALSE
**-8.31%**	Adrenergic, Alpha 1B	[3H]-7-MeOxy-Prazosin	FALSE
**6.75%**	Adrenergic, Alpha 2A (h)	[3H]MK-912	FALSE
**-14.09%**	Adrenergic, Alpha 2B	[3H]MK-912	FALSE
**6.28%**	Adrenergic, Alpha 2C (h)	[3H]MK-912	FALSE
**11.21%**	Adrenergic, Beta 1 (h)	[125I] (-) Iodocyanopindolol	FALSE
**-8.25%**	Adrenergic, Beta 2 (h)	^{125}I-Iodo-cyanopindolol	FALSE
**-5.87%**	Dopamine Transporter	[3H]WIN 35,428	FALSE
**-2.94%**	Dopamine, D1 (h)	[3H]-SCH23390	FALSE
**-21.17%**	Dopamine, D2s (h)	[3H]-Raclopride	FALSE
**-16.93%**	Dopamine, D3	[3H]7-OH-DPAT	FALSE
**-2.44%**	Dopamine, D4.4 (h)	[3H]-YM-09151-2	FALSE
**-2.66%**	GABA A, Agonist Site	[3H]GABA	FALSE
**11.84%**	GABA A, BDZ, alpha 1 site	[3H]Flunitrazepam	FALSE
**-2.43%**	GABA-B	[3H]CGP 54626A	FALSE
**-1.26%**	Glutamate, AMPA Site (Ionotropic)	[3H]AMPA	FALSE
**-6.93%**	Glutamate, Kainate Site (Ionotropic)	[3H]Kainic acid	FALSE
**5.46%**	Glutamate, MK-801 Site (Ionotropic)	[3H]MK-801	FALSE
**5.51%**	Glutamate, NMDA Agonist Site (Ionotropic)	[3H]CGP 39653	FALSE
**7.71%**	Glutamate, NMDA, Phencyclidine Site (Ionotropic)	[3H]TCP	FALSE
**4.34%**	Glutamate,NMDA,Glycine (Stry-insens Site) (Ionot	[3H]-MDL-105,519	FALSE
**-7.63%**	Glycine, Strychnine-sensitive	[3H]Strychnine	FALSE
**15.56%**	Histamine, H1	[3H]Pyrilamine	FALSE
**6.50%**	Histamine, H2	[125I]-Aminopotentidine	FALSE

**5.85%**	Histamine, H3	[3H]N-a-MeHistamine	FALSE
**-11.73%**	Muscarinic, M1 (hr)	[3H]Scopolamine, N-Methyl	FALSE
**-3.18%**	Muscarinic, M2 (h)	[3H]Scopolamine, N-Methyl	FALSE
**-4.77%**	Muscarinic, M3 (h)	[3H]Scopolamine, N-Methyl	FALSE
**-6.69%**	Muscarinic, M4 (h)	[3H]Scopolamine, N-Methyl	FALSE
**2.24%**	Muscarinic, M5 (h)	[3H]Scopolamine, N-Methyl	FALSE
**93.33%**	**Nicotinic, Neuronal (α-BnTx insensitive)**	**[3H]Epibatidine**	**TRUE**
**23.72%**	Norepinephrine Transporter	[3H]Nisoxetine	FALSE
**-2.12%**	Opioid, Delta 2 (h)	[3H]-Naltrindole	FALSE
**-6.43%**	Opioid, Mu (h)	[3H]-Diprenorphine	FALSE
**7.03%**	Serotonin Transporter	[3H]Citalopram, N-Methyl	FALSE
**17.60%**	Serotonin, 5HT1A (h)	[3H]-8-OH-DPAT	FALSE
**9.99%**	Serotonin, 5HT1D	[3H]5-CT	FALSE
**12.43%**	Serotonin, 5HT2A	[3H]Ketanserin	FALSE
**-2.21%**	Serotonin, 5HT2C	[3H]Mesulergine	FALSE
**7.50%**	Serotonin, 5HT3	[3H]GR 65630	FALSE
**-16.64%**	Serotonin, 5HT4	[3H]GR 113808	FALSE
**1.24%**	Serotonin, 5HT5A (h)	[3H]-LSD	FALSE
**8.87%**	Serotonin, 5HT6 (h)	[3H]-LSD	FALSE
**-2.10%**	Serotonin, 5HT7 (h)	[3H]LSD	FALSE
**-0.52%**	Sigma 1	[3H]-(+)-Pentazocine	FALSE
**-0.65%**	Sigma 2	[3H]-DTG	FALSE
**-3.91%**	Calcium Channel, Type L (Dihydropyridine Site)	[3H]Nitrendipine	FALSE
**-0.60%**	Calcium Channel, Type N	[125I]-Conotoxin GVIA	FALSE
**6.83%**	GABA, Chloride, TBOB Site	[3H]TBOB	FALSE
**52.12%**	**Potassium Channel, ATP-Sensitive**	**[3H]Glibenclamide**	**TRUE**
**-2.57%**	Potassium Channel, Ca2+ Act., VI	[125I]Apamin	FALSE
**14.46%**	Potassium Channel, I[Kr] (hERG) (h)	[3H]Astemizole	FALSE
**6.91%**	Sodium, Site 2	[3H]Batrachotoxin A 20-a-Benzo	FALSE

**-10.86%**	Nitric Oxide, NOS (Neuronal-Binding)	[3H]NOARG	FALSE
**29.86%**	Leukotriene, LTB4 (BLT)	[3H]LTB4	FALSE
**-8.26%**	Leukotriene, LTD4 (CysLT1)	[3H]LTD4	FALSE
**3.14%**	Thromboxane A2 (h) [SQ 29,548]	3H SQ 29,548	FALSE
**24.85%**	Angiotensin II, AT1 (h)	[125I]-(Sar1-Ile8) Angiotensin	FALSE
**32.29%**	Bradykinin, BK2	[3H]Bradykinin	FALSE
**8.93%**	Endothelin, ET-A (h)	[125I] Endothelin-1	FALSE
**2.10%**	Neurokinin, NK1	[3H]Substance P	FALSE
**2.16%**	Neuropeptide, NPY2 (h)	[125I]-PYY	FALSE
**21.55%**	Esterase, Acetylcholine	Acetylthiocholine	FALSE
**e12.00%**	Phosphodiesterase, PDE4A1A (h)	Fluorescent cyclic AMP	FALSE
**-3.00%**	Phosphodiesterase, PDE5A1 (h)	Fluorescent cyclic GMP	FALSE
**-5.00%**	Kinase, Protein, PKA (h)	Fluorescein-labeled peptide	FALSE
**-1.00%**	Kinase, Protein, PKCa (h)	Fluorescein-labeled peptide	FALSE

TC299423 inhibited >50% of the reference radioligand for two sites: Neuronal nAChRs and ATP-sensitive potassium channels. 1µM TC299423 inhibited 93.33% of the [^{3}H]-epibatidine binding at nAChRs and 52.12% of the [^{3}H]-Glibenclamide binding at the ATP-sensitive potassium channels.

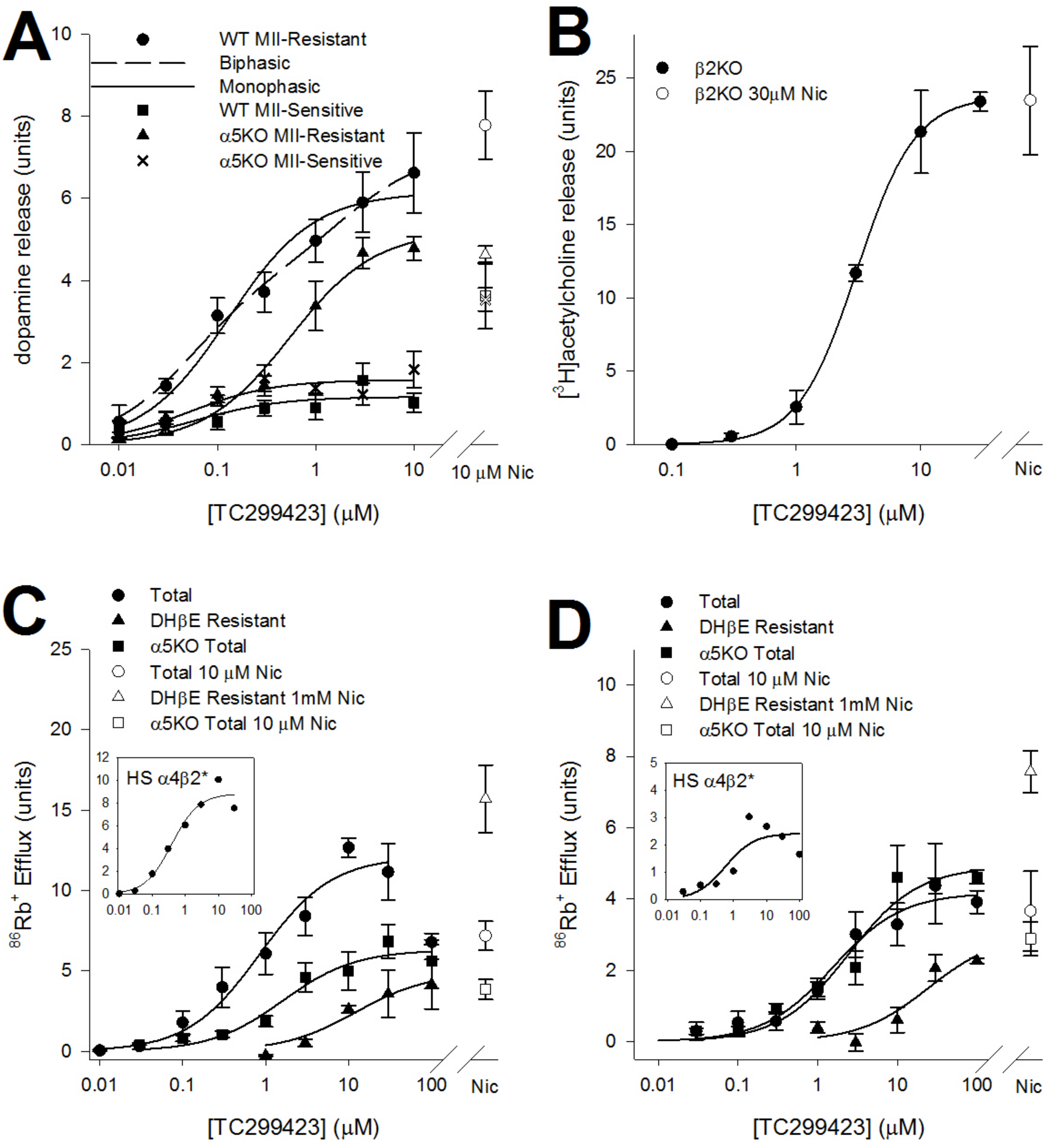

S1 Figure. Functional assays for measuring TC299423 agonist activity at various nAChRs. All points are mean ± SEM. **A)** Closed symbols show α-CtxMII-resistant [^{3}H]-dopamine release and α-CtxMII-sensitive [^{3}H]-dopamine release from WT and α5KO striatal synaptosomes. Open symbols show [^{3}H]-dopamine release evoked by 10 μM nicotine for the matching closed symbols. **B)** Measurement of functional α3β4*-nAChR by [^{3}H]-ACh release from β2KO IPN synaptosomes. Curve fit EC_{50} and efficacy values for (**A)** and (**B)** are shown in Table 1 (see Results). **C)** Activity of TC299423 on α4β2* measured by high-sensitivity $^{86}Rb^+$ efflux from WT

and α5KO thalamic synaptosomes. 2 μM DHβE was used to isolate the high-sensitivity and low-sensitivity responses. **D)** Activity of TC299423 on α4β2* measured by high-sensitivity $^{86}Rb^{+}$ efflux from WT and α5KO cortical synaptosomes. As in C, 2 μM DHβE was used to distinguish between high-sensitivity and low-sensitivity nAChRs. Calculated EC_{50} and efficacy values for **(C)** and **(D)** are presented in Table 2 (see Results).
